# Limited Impact of Column Chemistry and Length on Proteome Coverage Under High-Speed DIA

**DOI:** 10.1016/j.mcpro.2026.101609

**Published:** 2026-06-23

**Authors:** Alicia-Sophie Schebesta, Kathrin Korff, Ericka C.M. Itang, Vincent Albrecht, Philipp E. Geyer, Johannes B. Mueller-Reif

**Affiliations:** 1 Department of Proteomics and Signal Transduction, Max Planck Institute of Biochemistry, Martinsried, Germany; 2 ions.bio GmbH, Martinsried, Germany

## Abstract

The evolution of mass spectrometry (MS)-based proteomics has been driven by continuous technological advances in sample preparation, liquid-phase separations, instrumentation, and data acquisition. Chromatographic performance has been recognized as a contributing factor to identification depth, particularly on earlier-generation MS platforms. Recent advances in MS sampling speed and sensitivity now raise the question of how strongly chromatographic quality continues to determine overall proteome coverage. We investigate how column chemistry and length influence proteome coverage and chromatographic selectivity under modern data-independent acquisition conditions, and whether traditional optimization priorities still apply. Spanning a matrix of experiments with five distinct stationary phases, including C18 chemistries, C8, and Phenyl-Hexyl, across eight column lengths (40–140 mm), we evaluate protein identification performance using data-independent acquisition on the Orbitrap Astral mass spectrometer. Despite differences in stationary-phase chemistry and column length, we observed remarkably convergent proteome coverage metrics. All C18 and C8 phases consistently achieved over 150,000 precursor- and approximately 9000 protein group identifications, regardless of column length variations. While retention fingerprints persisted across chemistries, these chromatographic differences did not translate into meaningful variations in proteome coverage under high-speed acquisition conditions at 200 Hz. Within the range of modern sub-2 μm reversed-phase materials tested, identification depth showed limited dependence on column chemistry and length, suggesting that for state-of-the-art stationary phases, method development priorities may increasingly favor operational robustness, throughput, and reproducibility over traditional separation optimization.

## Highlights

• Comprehensive evaluation of five column chemistries across eight lengths reveals convergent proteome coverage despite substantial chromatographic differences.
• All C18 and C8 phases achieve >150,000 precursors and ∼9000 proteins regardless of column length under high-speed DIA acquisition.
• Distinct selectivity fingerprints persist across chemistries, with unique precursor populations identified by different stationary phases.

## Graphical Abstract

**Figure:**
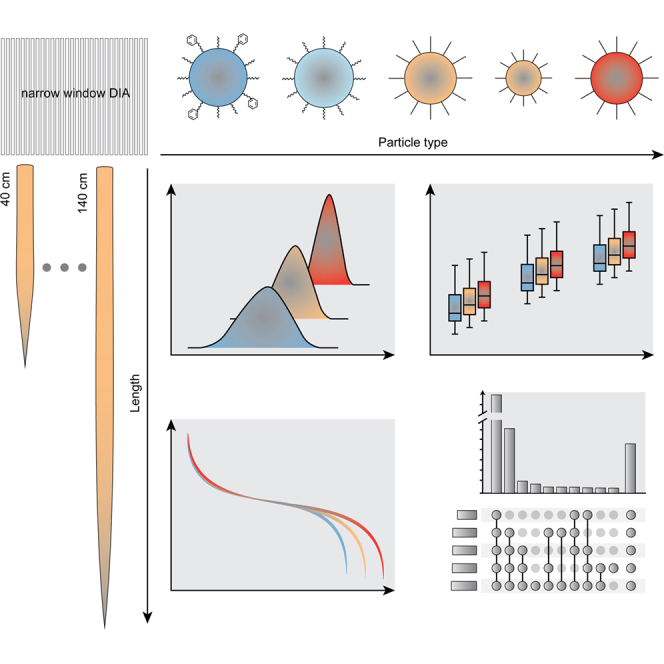

## In Brief

Chromatographic performance has traditionally been viewed as a key factor limiting proteome depth in mass spectrometry-based proteomics. We evaluated whether this paradigm persists under modern high-speed acquisition by varying column chemistry and length. Our findings indicate that analytical priorities have shifted from separation optimization toward operational robustness and throughput, reshaping method development in contemporary proteomics.

LC-MS/MS is the central analytical platform for proteome analysis, with reversed-phase high-performance liquid chromatography widely used for peptide separation (1, 2, 3, 4, 5, 6). Traditional proteomics workflows have long relied on capillary columns, typically 75 to 150 μm inner diameter, 15 to 50 cm length, and sub-2 μm packing material, which have been operated with capillary to nanoflow systems to maximize sensitivity. This general setup has remained largely consistent for nearly 2 decades, even as mass spectrometer performance has constantly advanced.

The principles of reversed-phase chromatography suggest that several parameters influence separation efficiency, including column chemistry, length, gradient conditions, and flow rate. Different stationary phases, such as C8 and C18 alkyl chains, polar-embedded groups, or Phenyl-Hexyl ligands, modulate retention primarily through hydrophobic interactions, with secondary contributions from features such as aromatic π–π interactions or hydrogen bonding. These differences can affect peptide retention behavior selectivity, particularly analytes with challenging physicochemical properties. C18 phases typically provide the strongest hydrophobic retention and broadest applicability for peptide separations, while C8 phases offer reduced hydrophobic character that can prevent excessive retention of highly hydrophobic peptides and improve elution behavior. Classic selectivity studies have shown that stationary-phase chain structure, bonding density, and ligand architecture govern not only hydrophobic retention but also shape-selective interactions, whereby more rigid or aromatic analytes interact differently with ordered or sterically accessible bonded phases (7). Phenyl-Hexyl phases provide aromatic selectivity *via* π–π interactions in addition to hydrophobic retention and are not commonly used in proteomics workflows. Their inclusion in this study served to extend the range of chromatographic selectivity tested and to provide a chemically distinct reference point against which the performance of standard reversed-phase materials could be assessed. To separate these selectivity effects from kinetic performance, it is important to recognize that particle morphology, pore structure, and surface area also shape chromatographic behavior by controlling mass-transfer kinetics and peak width, and modern particle technologies are designed to minimize band broadening and improve efficiency. Increasing column length can, under many conditions, contribute to higher apparent peak capacity (8, 9, 10, 11). However, this often comes at the cost of increased backpressure, extended runtimes, and diminishing gains under long gradient conditions. Historically, the limited acquisition speed of early-generation mass spectrometers made chromatography a key factor in precursor identification performance. With instruments acquiring up to ∼40 MS/MS scans per second, chromatographic peak shape and width were still shown to meaningfully impact identification depth, as demonstrated by Shishkova *et al*., who described peptide separations as "a largely overlooked… primary obstacle to achieving rapid, whole-mammalian-proteome analysis” (10, 11, 12, 13). From a mass spectrometry (MS) perspective, narrow chromatographic peaks improve sampling efficiency at low MS/MS rates by concentrating analyte signal into fewer scans, thereby increasing signal intensity and reducing coelution with other species. Under these constraints, careful optimization of column material, length, and particle size was essential (14).

In recent years, advances in MS instrumentation, such as the Orbitrap Astral with MS/MS rates of 200 Hz, have markedly increased acquisition speed and sensitivity (15, 16, 17). Modern platforms can routinely quantify over 10,000 proteins in 30 min runs and ∼7000 proteins in only 5 min using data-independent acquisition (DIA) (16). These capabilities have substantially reduced the reliance on hour-long gradients for deep proteome analysis. What was previously achievable only with extended LC separations can now be accomplished on the minute scale, enabling high-throughput applications such as population proteomics, interaction profiling, drug-target screening, and single-cell analyses. These improvements were driven not only by advances in MS hardware but also by improvements in LC technology and computational workflows (18, 19, 20).

Here, we ask how column chemistry and length influence both proteome coverage and chromatographic selectivity under modern high-speed DIA conditions, and whether traditional optimization priorities still apply. These advances prompted us to ask whether optimizing chromatographic parameters, such as column chemistry, length, particle size, gradient duration, or flow rate still has the same impact on identification performance under modern DIA conditions. We hypothesized that improvements in acquisition speed and sensitivity may have reduced the practical influence of chromatographic resolving power on proteome coverage.

To examine this, we comprehensively evaluated the impact of two key chromatographic variables, stationary-phase chemistry and column length, under tightly controlled acquisition conditions on a state-of-the-art DIA-MS platform. The resulting experimental matrix, five stationary phases spanning diverse retention behavior and eight column lengths from 40 to 140 mm was originally designed as a practical benchmarking dataset for column selection. Its breadth and consistency also enabled a retrospective assessment of whether chromatographic variation translates into meaningful differences in proteome coverage. To establish the boundaries of this behavior, we further tested representative conditions across varying sample amounts, biological matrices, and gradient lengths.

In summary, this study investigates the impact of column chemistry and length on proteome coverage and retention behavior in high-speed DIA workflows, thereby defining their current relevance in LC-MS-based proteomics applications.

## Experimental Procedures

### Experimental Design

#### HeLa Cell Cultivation and Lysate Preparation

HeLa cells (S3 subclone, ATCC) were maintained in Dulbecco's modified Eagle's medium supplemented with 10% fetal bovine serum, 20 mM glutamine, and 1% penicillin-streptomycin at 37 °C in a humidified 5% CO2 atmosphere. *Mycoplasma* testing was performed regularly to ensure culture sterility.

Cells were grown to approximately 80% confluence before harvesting. Following trypsinization with 0.25% trypsin/EDTA solution, cells were collected and transferred to 15 ml conical tubes. Cell pellets were washed twice using cold Tris-buffered saline and recovered by centrifugation at 200 *g* for 10 min. After removing the supernatant, cell pellets were immediately frozen in liquid nitrogen and stored at −80 °C for subsequent analysis.

Cell pellets were lysed using the PREOMICS lysis buffer (PreOmics, Martinsried). The lysis protocol involved heating samples at 95 °C for 15 min with continuous agitation at 1500 rpm, followed by probe sonication using a Bioruptor Plus sonication device with Minichiller 300 cooling system (Diagenode, Cat. No. B01020014) with short pulses (10 cycles of 5 s on/off at 20% amplitude). Cellular debris was removed by high-speed centrifugation for 5 min.

#### K562 Cell Lysate Preparation

MS Compatible Human Protein Extract from K562 cells (Promega, Cat. No. V6941, 1 mg) was used as a standardized protein source for dilution series experiments. The commercial lysate was reconstituted in lysis buffer containing 100 mM Tris, 40 mM chloroacetamide (CAA), and 10 mM Tris(2-carboxyethyl)phosphine, and heated at 95 °C for 10 min with continuous agitation at 1500 rpm.

#### Human Plasma Preparation

Peripheral blood was collected from a healthy adult donor into 10 ml EDTA tubes. Immediately after collection, tubes were gently inverted three times to ensure anticoagulant mixing, followed by centrifugation at 3,000*g* for 30 min. The plasma was collected and stored at −80 °C. Samples were stored for approximately 6 months before use and underwent a single freeze-thaw cycle. No additional anticoagulants, preservatives, or protease inhibitors were added beyond the EDTA present in the collection tubes.

Sample collection was conducted with written informed consent from the participant and was approved by the ethics committee of the Max Planck Society for the Advancement of Science (Reg. No. 2025_35). The study protocol adhered to the principles of the Declaration of Helsinki.

Plasma samples were prepared by adding 24 μl of buffer (100 mM Tris, 40 mM CAA, 10 mM Tris(2-carboxyethyl)phosphine) to 1 μl of plasma. The mixture was heated at 95 °C for 10 min with continuous agitation at 1500 rpm.

#### Mouse Liver Preparation

Animals used were bred for scientific purposes, and the research in this project does not involve experiments on animals (as defined by law). All animals were sacrificed by CO_2_ euthanasia prior to removal of organs in accordance with the European Commission Recommendations for the euthanasia of experimental animals (Part 1 and Part 2). Breeding, housing, and euthanasia of the animals are fully compliant with all German (*i.e*., German Animal Welfare Act) and EU (*i.e*., Directive 2010/63/EU) applicable laws and regulations concerning care and use of laboratory animals.

Frozen mouse liver tissue was homogenized in 500 μl of lysis buffer (60 mM triethylammonium bicarbonate, 40 mM CAA, 10 mM DTT) using an IKA T10 basic homogenizer (IKA-Werke GmbH & Co KG). Following homogenization, n-dodecyl β-D-maltosid was added to a final concentration of 0.01%. The homogenate was centrifuged at 1000*g* for 2 min, and the supernatant was transferred to a low-binding tube. Samples were sonicated using a Bioruptor Plus sonication device (15 cycles of 30 s on/off) and centrifuged again at 1000*g* for 2 min. Protein concentration was measured by NanoDrop One spectrophotometry (Thermo Fisher Scientific). Samples were diluted to a final concentration of 100 μg in 90 μl, and 10 μl of 100% acetonitrile was added (final concentration 10%). The mixture was heated at 75 °C for 60 min at 750 rpm, allowed to cool briefly, and centrifuged at 1000*g* for 2 min before transfer to a new tube.

#### Arabidopsis Tissue Preparation

*Arabidopsis thaliana* leaf tissue was ground to a fine powder in liquid nitrogen using a mortar and pestle. The ground tissue was resuspended in PreOmics lysis buffer (Cat. No. P.O.00001, PreOmics, Martinsried, Germany) for reduction of disulfide bridges and cysteine alkylation. Protein denaturation was performed at 95 °C for 10 min with continuous agitation. Samples were sonicated using a Bioruptor Plus sonication device (15 cycles of 30 s on/off).

#### Proteolytic Digestion and Peptide Purification

For proteolytic digestion, both Lys-C and trypsin were added at a 1:100 enzyme-to-protein ratio (w/w) and incubated overnight at 37 °C with gentle agitation at 1500 rpm. The digestion reaction was terminated by acidification with 1% TFA. Peptides were purified using styrene-divinylbenzene-reversed phase sulfonate solid-phase extraction with in-house packed StageTips containing 3M Empore styrene-divinylbenzene-reversed phase sulfonate extraction disks (47 mm diameter, polystyrenedivinylbenzene reversed-phase sulfonate) and reconstituted in 0.1% formic acid with 2% acetonitrile for LC-MS/MS analysis.

#### Data Acquisition

All columns used in this study were 75 μm inner diameter capillaries pulled and packed by Dr. Maisch GmbH. Five different stationary phases were systematically evaluated across eight column lengths (40, 50, 60, 70, 80, 100, 120, and 140 mm) to assess the impact of column chemistry and length on separation performance.

The following stationary phases and particle sizes were tested: Exsil Mono 100 C18 (1.35 μm particles, Cat. No. 6136973), Reprosil Saphir 100 C18 (1.3 μm particles, Cat. No. ra113.9e), Reprosil Saphir 100 C18 (1.5 μm particles, Cat. No. ra115.9e), Reprospher 100 C8 (1.8 μm particles, Cat. No. rs118.8e), and Reprospher 100 Phenyl-Hexyl (1.8 μm particles, Cat. No. rs118.ph). Physicochemical properties of the stationary phases, including carbon load, bonding type, and particle size distribution, are provided in Supplemental Table S1. All columns featured integrated electrospray emitter tips created by laser pulling. No column oven was used; columns were operated at ambient laboratory temperature. As a consequence, minor retention time variability attributable to ambient temperature fluctuations cannot be excluded and should be considered when interpreting retention time comparisons. Not all stationary phases could be evaluated at all eight column lengths. Individual columns at longer lengths were unavailable because pressure limits of the LC were reached, resulting in different numbers of evaluated lengths per stationary phase. Configurations tested per chemistry: EM (40, 50, 60, 70, 80, 100, and 120 mm), RS13 (40–140 mm, all eight lengths), RS15 (40–140 mm, all eight lengths), C8 (40, 50, 60, 70, 80, 100, and 120 mm), PH (40, 50, 60, 70, and 80 mm).

Tryptic digests were injected for LC-MS/MS analysis using a Thermo Scientific Vanquish Neo LC system coupled to an Orbitrap Astral mass spectrometer (Thermo Fisher Scientific) (17, 21). For HeLa, plasma, mouse liver, and Arabidopsis samples, 200 ng of sample were injected per run. For K562 samples, a dilution series was analyzed with injection amounts of 50 ng, 5 ng, 1 ng, 0.5 ng, and 0.1 ng per run. The LC system was operated at a maximum pressure of 1500 bar with direct injection capability. Mobile phases consisted of 0.1% formic acid in water (A) and 0.1% formic acid in acetonitrile (B). For peptide separation, a 20-min gradient was employed at a flow rate of 0.5 uL/min, starting at 2% B, with a linear increase to 31% B, followed by a ramp to 90% B for column wash and reequilibration to initial conditions. Mass spectrometric analysis was performed in DIA mode with the ion source operated in positive polarity with a spray voltage of 1900 V, ion transfer tube temperature of 280 °C, and RF lens value set to 40%. Full MS scans were acquired in the Orbitrap analyzer at a resolution of 240,000 over a scan range of 380 to 980 m/z with a normalized automatic gain control (AGC) target of 500% (absolute AGC value of 5.00e6). The liquid chromatography mode was selected with an expected LC peak width of 10 s, and advanced peak determination was enabled. The default charge state was set to 2, and Orbitrap lock mass correction was turned off. For DIA analysis, the precursor mass range was set to 380 to 980 m/z, divided into 300 scan events with an isolation window of 2 m/z and window placement optimization enabled. MS2 spectra were acquired using HCD with a normalized collision energy of 25%. Fragment ions were detected using the Astral detector with a scan range of 150 to 2000 m/z. The normalized AGC target was set to 500% (absolute AGC value of 5.00e4) with a maximum injection time of 3 ms. Data were collected in centroid mode for MS2 scans with positive polarity. Quality control samples were analyzed regularly throughout the analytical sequence to monitor system performance and stability.

### Statistical Rationale

This study is a technical benchmarking investigation designed to evaluate chromatographic performance rather than biological variability. Accordingly, all experiments were performed using single biological preparations: HeLa lysate was prepared from 1 cell culture batch, K562 lysate was obtained from a single commercial preparation (Promega), human plasma was collected from one healthy donor, mouse liver tissue was harvested from one animal, and Arabidopsis tissue was prepared from a single harvest. For each experimental condition (column chemistry × column length), four technical replicates were acquired, with the first replicate excluded as a column-conditioning run, yielding three replicates for analysis. This design was chosen to isolate analytical variability attributable to chromatographic parameters while maintaining practical feasibility across almost 40 column conditions tested. Reproducibility was assessed using CVs at both protein and precursor levels; median CVs below 5% at the protein level confirmed robust technical precision across conditions.

#### Data Analysis/Spectral Search

Raw MS files were converted to mzML format using ThermoRawFileParser (v4.3, Thermo Fisher Scientific) with format and metadata parameters set to 1 and 0, respectively. File conversion was performed in parallel on a high-performance computing cluster for efficient processing.

Spectral searches were conducted using DIA-NN (version 2.0) (22) against the UniProt human Swiss-Prot reference proteome database, including isoforms (version 19.02.2025, 42,519 protein entries). An in-silico spectral library was generated from the FASTA sequences incorporating oxidation of methionine and N-terminal acetylation as variable modifications. Trypsin specificity was set with up to two missed cleavages allowed. Mass accuracy tolerances were set to 10 ppm for both MS1 and MS2 levels, with a scan window parameter of 7.

Search parameters included match-between-runs functionality using the "--usequant" and "--reanalyse" flags, along with peak centering, smart profiling, retention time profiling, and relaxed protein inference settings.

False discovery rate control was applied at 1% at the precursor (peptide-spectrum match) level using a target-decoy approach, at 1% library match confidence and 1% protein group assignment. Precursors were defined as unique combinations of peptide sequence, charge state, and modifications. For downstream quantitative analysis, the DIA-NN reports table was used and further filtered (Entries were required to satisfy Q.Value < 0.01 (precursor-level FDR), Lib.Q.Value ≤ 0.01 (library match confidence), and Lib.PG.Q.Value ≤ 0.01 (protein group assignment confidence)). Protein quantification integrated both MS1 and MS2 information, and interference removal was not applied.

Chromatographic peak capacity was calculated according to:

$$n_{c}=1+\frac{t_{g}}{{1.7*w}_{h}}$$

where t_g_ is the gradient time of 20 min and w_h_ is the peak width at half-height (FWHM).

#### Bioinformatics Analysis

All data processing and analysis steps were performed in the Python programming environment (v.3.12.10) using custom analysis scripts. Data processing was conducted using pandas (v2.2.3) for manipulation of proteomics data matrices and NumPy (v2.2.5) for numerical operations, while visualization and statistical analyses employed SciPy (v1.15.3), matplotlib (v3.10.3), and seaborn (v0.13.2). Although four technical replicates were measured for each column condition, only three replicates were included in the final analysis, with the first run of each column excluded from the dataset. Visualization of raw files was conducted using the alpharaw package (https://github.com/MannLabs/alpharaw).

## Results

**Fig. 1:**
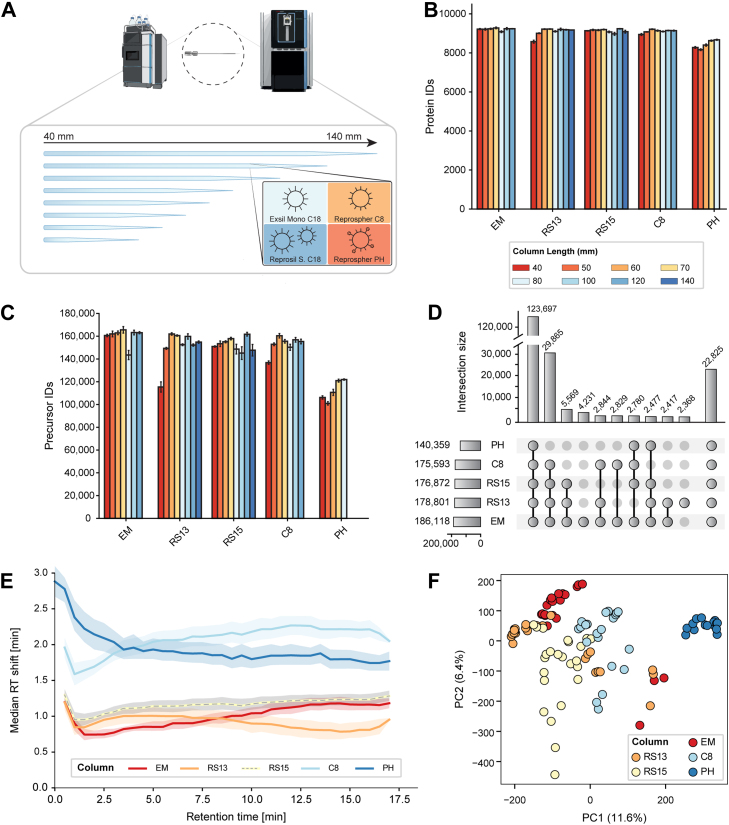
**Column performance comparison across chemistries and lengths.***A*, schematic overview of experimental design testing five stationary phases across eight lengths. *B*, protein group identifications across column materials and lengths. Configurations tested per chemistry: EM (40, 50, 60, 70, 80, 100, and 120 mm), RS13 (40–140 mm, all eight lengths), RS15 (40–140 mm, all eight lengths), C8 (40, 50, 60, 70, 80, 100, and 120 mm), PH (40, 50, 60, 70, and 80 mm). *C*, precursor identifications across column materials and lengths. *D*, UpSet plot showing precursor overlap between column chemistries at 70 mm. Only the top intersections are shown; all remaining overlaps and unique sets are aggregated in the final bar. *E*, median retention time differences (ΔRT = RT_80_ – RT_40_) plotted as a function of retention time from 40 mm columns. *F*, PCA of precursor intensities colored by column chemistry. Missing values were imputed using k-nearest neighbor imputation (k = 5). Intensities were standardized (mean = 0, variance = 1) prior to principal component analysis. EM, Exsil Mono C18 (1.35 μm); RS13, Reprosil Saphir C18 (1.3 μm); RS15, Reprosil Saphir C18 (1.5 μm); RT, retention time; PCA, principal component analysis.

To benchmark the performances of various columns, we measured a tryptic digest of HeLa, prepared from a single biological preparation, in triplicate across an experimental matrix of five different column chemistries (Exsil Mono C18 (1.35 μm; EM), Reprosil Saphir C18 (1.3 μm; RS13), Reprosil Saphir C18 (1.5 μm; RS15), Reprospher C8 (1.8 μm; C8), and Reprospher Phenyl-Hexyl (1.8 μm; PH)) and eight column lengths (40, 50, 60, 70, 80, 100, 120, 140 mm) (Fig. 1*A*). This dataset formed the basis for assessing how chromatographic differences relate to proteome coverage under fast DIA acquisition.

Not all chemistries could be tested at all eight lengths. RS13 and RS15 were evaluated across the full-length range (40–140 mm), while individual longer columns for EM, C8, and PH were unavailable due to column failures during handling (see Fig. 1 legend for details). Comparisons of identified protein groups (Fig. 1*B*) and precursors (Fig. 1*C*) reveal broadly consistent performance across all conditions. The EM columns yield the highest identifications with an average of 9200 protein groups and over 160,000 precursors, followed closely by RS13, RS15, and C8, each exceeding 150,000 precursors and ∼9100 protein groups. In contrast, PH consistently identifies the lowest number of proteins (∼8400) and precursors (∼110,000).

While protein identifications remain largely unaffected by column length, precursor identifications show modest variation. The EM column demonstrates high performance across lengths, with only the 80 mm variant showing slightly lower performance. For RS13, identifications increase moderately from 40 to 60 mm before plateauing, whereas RS15 does not exhibit this trend. C8 displays gradually increasing precursor identifications with column length while maintaining stable protein-level performance. Despite its lower hydrophobic retention, C8 achieves identification levels comparable to the C18 chemistries, suggesting that under high MS sampling rates, differences in retention strength do not necessarily translate into reduced identification depth.

Although the identification outcomes are broadly similar, peptide elution behavior differs across column chemistries and lengths. Cumulative identifications across the gradient (Supplemental Fig. S1) show that over 80% of proteins are identified within the first third of the chromatographic run, with systematic shifts toward later elution as column length increases for all chemistries except PH. At the precursor level, the cumulative curves rise more gradually and plateau after approximately 15 min, reflecting progressive peptide separation along the gradient.

To determine whether these comparable identification numbers arise from detection of the same or distinct peptide populations, we next examined overlap patterns using 70 mm column data (Fig. 1*D*). More than 120,000 precursors (∼70%) are shared across all five columns, including the majority identified on PH. Additional overlap sets emerge between C8 and the C18 columns, and among the three C18 variants alone. Despite this extensive sharing, substantial unique identifications occur for EM and RS13 with 4231 and 2368 unique precursors, respectively. At the protein level, overlap patterns were similarly dominated by a large shared core across all chemistries (Supplemental Fig. S2).

To quantify how column length affects retention across chemistries, we compared retention time differences between 40- and 80-mm columns (Fig. 1*E*). PH and C8 exhibit substantially larger shifts (∼1.7–2.3 min), roughly two-fold higher than C18 chemistries (∼0.8–1.3 min). Distinct chemistry-specific patterns emerge across the gradient PH showed the largest shifts early in the gradient (∼2 min), which decreased toward later elution, whereas C8 displayed the opposite trend, with retention differences increasing toward late elution and exceeding 2 min. Among C18 phases, EM demonstrates minimal early sensitivity to column length (<0.7 min) that increases to >1 min at late retention times, indicating precursor retention becomes progressively more affected by column length. RS13 shows the inverse behavior with ∼1 min early differences that decrease over the gradient, meaning column length impact diminishes for late-eluting precursors. RS15 remains nearly constant (∼1.0–1.2 min) throughout. These analyses show that the effect of column length on retention time differs systematically across chemistries and across the gradient. Distributional analyses across retention-time bins are shown in Supplemental Fig. S3, and representative GAPDH precursor chromatograms are shown in Supplemental Fig. S4.

Beyond direct retention-time comparisons, principal component analysis of precursor intensities independently reveals systematic differences between column chemistries (Fig. 1*F* and Supplemental Fig. S5, *C* and *D*). Measurements cluster primarily by column chemistry, with PH forming a distinct group separated along PC1 (11.6% variance). EM and RS13 cluster closely together and separate from RS15 and C8 along PC2 (6.4% variance). This separation becomes even more pronounced at the protein level (Supplemental Fig. S5, *A* and *B*), where PH shows sharp separation along PC1 while C18 and C8 columns cluster closely together.

In summary, while identification depth remains consistent across column types and lengths, the underlying separation characteristics reveal meaningful differences. Column chemistry and length influence the elution behavior of shared precursors, with PH showing the most distinct retention patterns, while other columns, particularly EM, exhibit more uniform behavior across the gradient.

To ensure that these observations are not driven by technical variability, we evaluated quantitative reproducibility across technical replicates for each column chemistry and length (Supplemental Fig. S6). Detection frequency analysis revealed consistent performance, with more than 68% of all identified precursors detected across all three replicates (median 75%), while less than 17% appeared in only a single replicate. Quantitative precision was assessed through CVs at both protein and precursor levels. Protein-level CVs clustered around 5% for most columns, indicative of high-quality quantification, with the PH chemistry showing moderately elevated CVs consistent with its distinct chromatographic behavior. Precursor-level CVs were naturally higher, with occasional outliers likely reflecting inherent low-abundance variability rather than comprehensive performance issues. Chromatographic stability was good, with retention time standard deviations below 2 s for nearly all peaks, except for the RS13 40 mm and RS15 100 mm columns, which showed elevated variability consistent with previously noted irregularities. Collectively, these metrics demonstrate that despite chromatographic differences between columns, quantitative precision and technical reproducibility remain consistently high across all tested configurations.

Having established that proteome coverage is largely consistent across column chemistries and lengths and that quantitative performance is reproducible, we next examined chromatographic performance metrics to explore more subtle differences in separation behavior. We analyzed full width at half maximum (FWHM) as a measure of peak width and retention time correlations to characterize separation characteristics.

**Fig. 2:**
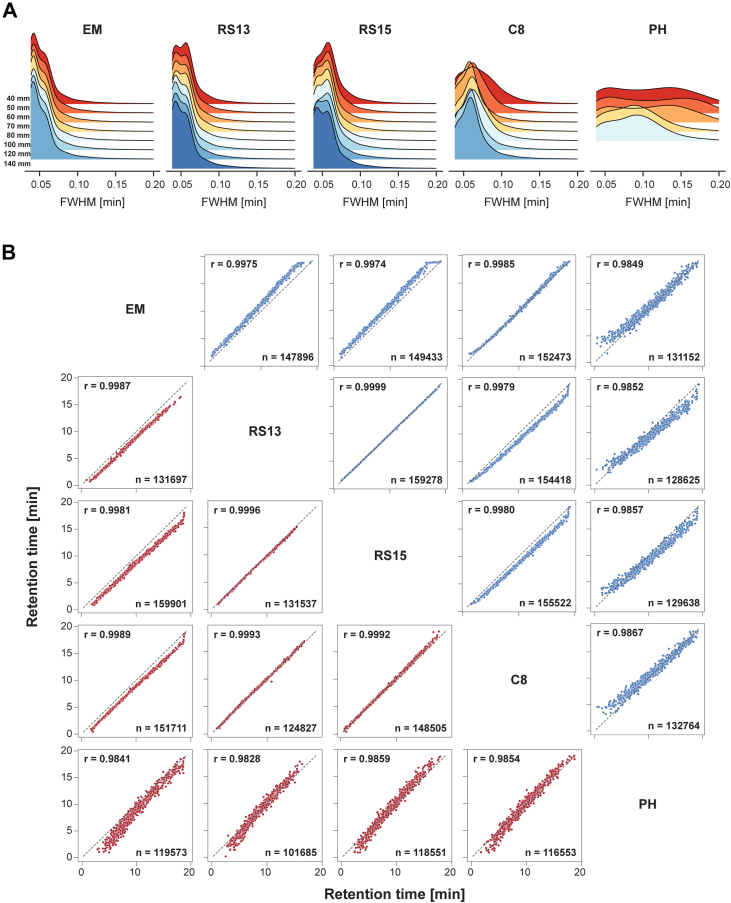
**Chromatographic performance comparison across column chemistries and lengths.***A*, FWHM distributions for each column type across different column lengths (40–140 mm), based on a 20-min gradient. Distributions include all identified precursors with FWHM <0.2 min. *B*, retention time correlation matrix for 40 mm (*red*) and 80 mm (*blue*) columns showing pairwise comparisons between column types. For visualization purposes, 0.5% of all data points are shown (randomly selected from all data points); all correlation calculations and statistical analyses were performed on the complete dataset. FWHM, full width at half maximum.

FWHM distributions across column chemistries (Fig. 2*A*) reveal that EM exhibits the sharpest peaks with a median FWHM of 3 s, followed closely by RS13 and RS15 at 3.2 and 3.4 s, respectively. C8 and PH columns show broader elution with median FWHMs of 3.6 and 6 s. Column length displays minimal impact on peak width for most chemistries under the steep gradient conditions used in this study, consistent with gradient-dominated peak width behavior in which gradient compression rather than column plate count governs the observed peak widths. C8 demonstrates more pronounced length dependence, with peaks becoming 13% sharper from 40 mm to 120 mm columns. PH exhibits the broadest peaks overall, lacking distinct modal distributions and showing only slight improvement with increased length.

Based on the measured baseline peak widths, the estimated peak capacities (Supplemental Fig. S7) were ∼235 for EM, ∼225 for RS13, ∼215 for RS15 ∼190 for C8, and ∼110 for PH, indicating higher separation performance of the C18-based phases. Despite some outlier values, the consistent median capacities across column lengths suggest that stationary-phase chemistry exerts a stronger influence on chromatographic performance than column length under these conditions.

In addition to peak width and separation capacity, retention time correlation analysis between shared precursors reveals high reproducibility across most column types (Fig. 2*B*). Correlations between EM and both RS variants are near perfect (r > 0.98), with precursors eluting slightly earlier on RS columns. This shift increases modestly with retention time but remains consistent and predictable.

C8 correlations are similarly strong, particularly for 80 mm columns, where retention times closely match EM, though late-eluting hydrophobic precursors show earlier elution on C8 compared to C18 chemistries, reflecting the expected lower retention capacity. PH demonstrates distinct behavior with slightly lower correlations (r = 0.98–0.99) and broader scatter patterns. Early-eluting precursors that appear at ∼5 min on other columns elute around ∼2 min on PH, indicating reduced retention of hydrophilic precursors. Conversely, hydrophobic precursors show enhanced retention on PH compared to other columns, creating a shifted separation profile with different retention characteristics.

To assess whether column length alters retention behavior within individual chemistries, we next examined retention time correlations across lengths for each stationary phase (Supplemental Figs. S8 and S9). Near-perfect correlations (r > 0.99) were observed for both RS13 and EM. Longer columns shift elution to later times in the gradient due to increased retention capacity, with this effect being more pronounced for EM, suggesting greater peptide length dependence for this chemistry.

These findings indicate that while overall performance remains comparable, column chemistry introduces differences in peak sharpness and retention behavior. Such variations, particularly for PH and C8 chemistries, could be advantageous for separating specific precursor populations with distinct physicochemical properties.

**Fig. 3:**
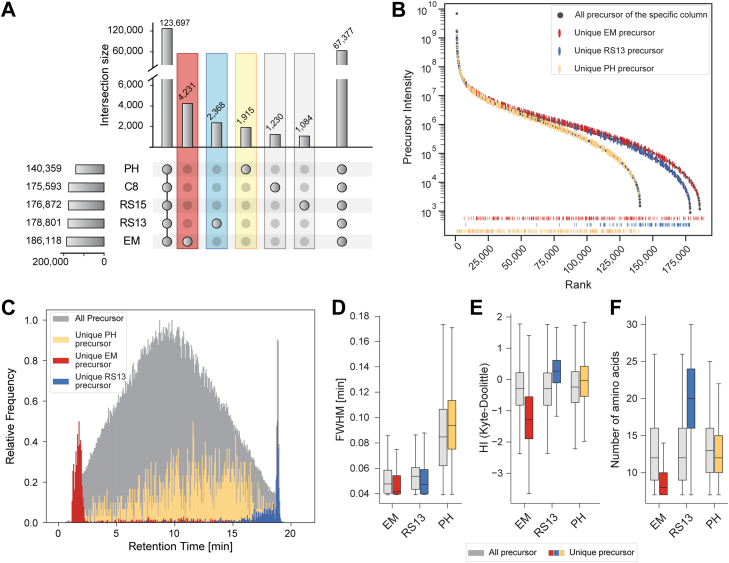
**Unique precursor characterization across column chemistries.***A*, modified UpSet plot showing precursors unique to each column (colored bars) along with the number shared across all five columns (*left*) and the sum of all remaining intersections (*right*). Intermediate overlaps between columns are not shown individually. *B*, shared rank-abundance plot for three selected columns: EM (*red*), RS13 (*blue*), and PH (*yellow*). The *lower panel* shows the distribution of unique precursors along the rank axis for each column; only 2% of all data points are shown. *C*, retention time distributions of all precursors (*gray*) and unique precursors (colored). Distribution of all precursors is normalized to one and distributions of the unique precursors is normalized to 0.5. *D*, FWHM comparison between all (*gray*) and unique (colored) precursors. *E*, hydrophobicity index (HI, Kyte-Doolittle scale) distributions comparing hydrophobicity between all (*gray*) and unique (colored) precursors. *F*, amino acid length distributions comparing peptide length between all (*gray*) and unique (colored) precursors. EM, Exsil Mono C18 (1.35 μm); RS13, Reprosil Saphir C18 (1.3 μm); FWHM, full width at half maximum.

Given the chemistry-dependent differences in retention behavior observed above, we next asked whether these translate into differences in the specific precursors detected. Although the majority of precursors are shared across all five column chemistries (Fig. 1D), a distinct subset is uniquely identified by individual columns, warranting further investigation (Fig. 3*A*). From a total of 201,902 identified precursors, 5% are detected exclusively with a single stationary phase, with the EM column accounting for 2.1% (4231 precursors) and RS13 contributing 1.2% of unique identifications. We define “column-unique” as detected only with that stationary phase (at the given length) and not with any other phase. To ensure interpretability, we applied a reproducibility cascade and retained only column-unique precursors present in all three technical replicates (Supplemental Fig. S10)

This stringent filtering yielded 1278 of 4231 precursors for EM (30%), 594 of 2368 for RS13 (25%), and 1131 of 1915 for PH (59%). PH demonstrates the highest reproducibility among unique precursors, reflecting the distinct retention behavior conferred by its alternative stationary phase chemistry.

Given the substantial differences observed, we focused the following analyses on the three columns exhibiting the most pronounced retention differences. Rank abundance analysis (Fig. 3*B* and Supplemental Fig. S11*A*) reveals that unique precursors span the full intensity range for EM and PH columns, demonstrating that these identifications are not merely low-abundance artifacts, but also include highly abundant species. In contrast, unique precursors from RS13 are predominantly ranked in the lower half of the abundance distribution.

Analysis of chromatographic elution profiles reveals distinct retention time signatures for unique precursors (Fig. 3*C* and Supplemental Fig. S11*B*). Unique EM precursors predominantly elute early in the gradient (1–3 min), while unique RS13 precursors elute exclusively at the gradient terminus (>15 min, with a pronounced peak at 19 min). This extreme elution behavior suggests that the bead size and chemistry enable the separation of precursors that are not identified on any other column type. In contrast, unique PH precursors exhibit a retention time distribution spanning the entire chromatographic range, though slightly shifted toward later elution compared to the overall precursor population. These distinct elution profiles indicate fundamental differences in physicochemical properties between unique precursor populations.

We therefore analyzed three key parameters to characterize these differences: FWHM as a measure of peak width, hydrophobicity index (HI, Kyte-Doolittle scale), and amino acid length. Peak width analysis (Fig. 3*D*) shows that PH columns generate broader chromatographic peaks with approximately double the FWHM compared to other columns. For both EM and RS13, uniquely identified precursors exhibit narrower peak widths than the overall precursor population, despite their opposing elution profiles.

Hydrophobicity index analysis (Fig. 3*E*) demonstrates substantial differences between unique precursor populations. While overall precursor populations show minimal differences across columns (median HI ≈ 0.0), unique EM precursors exhibit extreme hydrophilicity (median HI ≈ −1.1), reflecting their early elution and minimal retention by the C18 stationary phase. Conversely, unique RS13 precursors display elevated hydrophobicity (median HI ≈ +0.4), explaining their late elution and strong retention. Unique PH precursors show only marginally elevated values, consistent with their slight retention time shift.

Precursor length analysis (Fig. 3*F*) reveals comparable median lengths for overall precursor populations (12 amino acids for EM and RS13, 13 for PH). However, striking differences emerge for unique precursors. Unique EM precursors are substantially shorter (median eight amino acids), representing a one-third reduction compared to the overall population. Conversely, unique RS13 precursors are remarkably longer (median 20 amino acids), representing a >60% increase with complete separation between the two distributions. PH shows only a marginal difference between unique and total precursor lengths.

Together, these findings demonstrate that despite comparable overall performance metrics, different column chemistries provide distinct retention preferences for precursor subsets with specific physicochemical properties. This selectivity proves particularly valuable for applications requiring enhanced detection of very hydrophilic or hydrophobic peptides, highlighting the importance of tailoring column choice to specific analytical objectives.

To evaluate the boundaries of our findings and determine under which conditions column length may influence performance, we conducted additional experiments focusing exclusively on the EM C18 stationary phase. These experiments examined sample input amounts from 50 ng down to 100 pg, diverse biological sample matrices, and gradient lengths ranging from 5 to 60 min. Together, they expand the tested parameter space and characterize conditions under which chromatographic effects may become limiting.

**Fig. 4:**
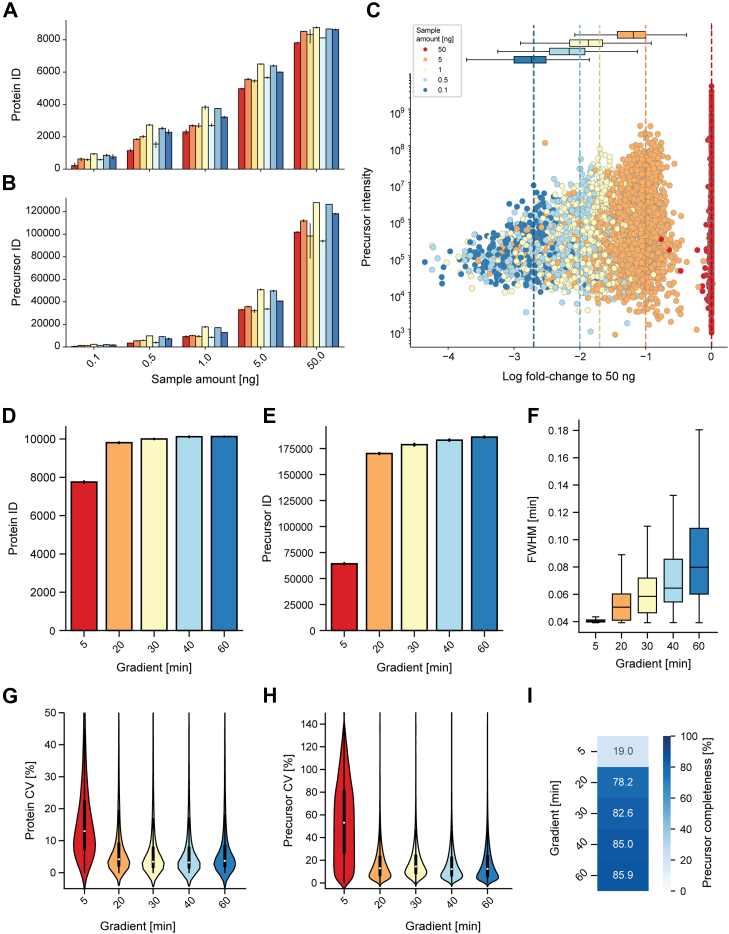
**Column performance across analytical conditions.***A*, protein group identifications across sample input amounts (100 pg to 50 ng) for K562 cell lysate on EM columns (40–120 mm). Bars show mean ± standard deviation (n = 3). *B*, precursor identifications across sample input amounts. Bars show mean ± standard deviation (n = 3). *C*, log_10_ fold-change of precursor intensities relative to 50 ng *versus* absolute intensity for the 70 mm column. *Dashed lines* indicate expected fold-changes. *D*, protein group identifications across gradient lengths (5–60 min) for 200 ng HeLa on a 70 mm EM column. Bars show mean ± standard deviation (n = 3). *E*, precursor identifications across gradient lengths. Bars show mean ± standard deviation (n = 3). *F*, FWHM distributions across gradient lengths. *G*, protein group CV distributions across gradient lengths. *H*, precursor CV distributions across gradient lengths. *I*, percentage of precursors detected in all three replicates across gradient lengths. EM, Exsil Mono C18 (1.35 μm); FWHM, full width at half maximum.

We first analyzed K562 cell lysate across five input amounts (50 ng, 5 ng, 1 ng, 500 pg, and 100 pg) using seven column lengths from 40 to 120 mm, each measured in triplicate. Identification depth decreased substantially with decreasing input (Fig. 4, *A* and *B*). At 50 ng, all columns identified more than 8000 protein groups and over 100,000 precursors, whereas at 100 pg identifications fell to approximately 500 proteins and 2500 precursors. Across this dynamic range, column length had minimal impact on identification depth under adequate sample loading (≥1 ng). From 40 to 120 mm, identification numbers varied by less than 5% at 50 ng, 5 ng, and 1 ng inputs. Only at the lowest inputs (100–500 pg) did the shortest 40 mm column show modestly reduced performance, suggesting that under severely sensitivity-limited conditions, longer columns may confer a small chromatographic advantage. The 80 mm column consistently underperformed and is considered a technical outlier, whereas the 70 mm column demonstrated particularly strong and stable performance. Quantitative analysis confirmed that log_2_ fold-changes relative to the 50 ng reference followed theoretical expectations across all sample amounts, with increased dispersion only at low inputs (Fig. 4*C* and Supplemental Fig. S12*A*). Individual precursors displayed clear linear behavior across the dilution series: for example, the GAPDH peptide VGVNGFGR^2+^ showed an excellent linear relationship between input amount and intensity across all column lengths (Supplemental Fig. S12*B*). Extending this to the full dataset, nearly all precursors exhibited R^2^ values between 0.99 and 0.999 regardless of column length (except the 80 mm outlier) (Supplemental Fig. S12*C*). These results demonstrate robust quantitative linearity over more than two orders of magnitude and indicate that column length does not impair accuracy or dynamic range under high-speed DIA acquisition.

To assess whether column length independence extends to more complex matrices, we analyzed Arabidopsis tissue, human plasma, and mouse liver at 200 ng input across all column lengths (Supplemental Fig. S12, *D* and *E*). Arabidopsis and mouse liver showed highly consistent identifications across columns, with only the shortest formats showing 5 to 15% reductions. Plasma, with its extreme dynamic range, showed more pronounced differences: the 40 mm column produced 20 to 25% fewer identifications than the 60 to 120 mm columns. Nevertheless, columns between 60 and 100 mm performed nearly equivalently even for plasma, suggesting that although high-dynamic-range matrices benefit moderately from increased chromatographic separation, the overall dependence on column length remains limited.

We next evaluated five gradient lengths (5, 20, 30, 40, and 60 min) using 200 ng HeLa digest on a 70 mm EM column, each measured in triplicate. Gradients were scaled proportionally without modifying MS acquisition settings, enabling direct comparison of chromatographic effects independent of instrument parameters. Protein and precursor identifications were largely stable across gradients from 20 to 60 min (Fig. 4, *D* and *E*). Protein identifications plateaued at ∼10,000 groups, while precursors increased only modestly (∼10%) as the gradient length tripled. In contrast, the 5-min gradient yielded 23% fewer proteins and 65% fewer precursors compared to longer gradients, but delivering the highest identification rate per unit time (Supplemental Fig. S13, *A* and *B*). Peak widths (FWHM) increased with gradient length (Fig. 4*F*), and sampling density (data points per peak) also increased (Supplemental Fig. S13*C*). However, these chromatographic improvements were not associated with improvements in quantitative precision. CVs were nearly identical for gradients from 20 to 60 min, with median CVs below 5% at the protein level and ∼12 to 13% at the precursor level (Fig. 4, *G* and *H*). Data completeness followed the same pattern: the 5-min gradient showed <20% completeness, whereas 20 to 60 min gradients achieved 78 to 86% (Fig. 4*I*). These results indicate that, under modern DIA acquisition, longer gradients do not appreciably improve identification depth or quantitative precision beyond ∼20 min.

Across variations in sample input, biological matrix complexity, and gradient duration, column length independence was preserved under most tested conditions, with well-defined boundary cases. For standard inputs (≥5 ng for cell lysates, ≥200 ng for complex tissues) and gradient lengths of 20 to 40 min, columns between 50 and 100 mm provide nearly equivalent performance across diverse matrices. Longer columns offer marginal benefits only under extremely sensitivity-limited conditions (<500 pg) or when analyzing very high-dynamic-range samples such as unfractionated plasma. Similarly, extending gradients beyond ∼30 min provides limited advantages under high-speed DIA, with diminishing returns in both depth and quantitative consistency. For modern routine proteomics applications, shorter columns (50–70 mm) paired with 20 to 30 min gradients offer performance comparable to traditional longer formats while reducing backpressure, simplifying method setup, and improving throughput. These findings provide practical guidance for laboratories seeking robust, efficient LC–MS workflows optimized for modern DIA platforms.

## Discussion

Our comprehensive evaluation of five column chemistries across eight lengths using state-of-the-art DIA-MS reveals how column chemistry and length shape both identification depth and chromatographic selectivity under modern DIA conditions, with distinct implications for each. While not originally designed as a hypothesis-driven investigation of chromatography-MS tradeoffs, the comprehensive nature of the column matrix enabled us to retrospectively evaluate how chromatographic characteristics relate to identification performance under modern DIA-MS. Despite clear differences in stationary phase chemistry and measurable variation in peak width, overall proteome coverage was highly similar; all C18 and C8 phases yielded >150,000 precursors and ∼9000 protein groups from HeLa digests. These performance metrics complement findings reported by Guzman *et al*. for nDIA methodology on human cell lysates (16).

While we observed clear and consistent differences in retention behavior and peak width between chemistries, these did not translate into substantial changes in overall identification depth under our acquisition conditions. This suggests that, in high-speed DIA workflows such as those enabled by the Orbitrap Astral MS, optimization of chromatographic performance has less impact on total proteome coverage than historically reported. We emphasize, however, that chromatography remains necessary for successful proteomic analysis; rather, our data show that within the range of modern, high-quality reversed-phase materials and practical column lengths, identification performance converges and chromatographic setups yield highly comparable results.

These observations differ from expectations rooted in earlier generations of LC-MS instrumentation, where peak width and resolving power were more tightly linked to identification depth (1, 2). Traditional workflows that often treated chromatography as a limiting factor for achieving greater proteome depth, drove the development of ultrahigh-pressure packing methods that could increase proteome depth by up to 35% (11, 23). At that time, even with faster MS instrumentation, it was argued that better chromatography remained essential to fully leverage instrument capabilities (13).

Our results suggest that under modern high-speed DIA conditions, the dependence of identification depth on chromatographic peak width is reduced, as >200 Hz acquisition rates enable comprehensive peptide identification through spectral deconvolution even when chromatographic peaks are broader (10, 16, 17). It is important to note, however, that the extent of this effect is likely influenced by instrument capabilities and acquisition strategy, and may differ under other analytical configurations. To our knowledge, this is the first structured evaluation under Astral DIA conditions, suggesting that within the range of modern sub-2 μm stationary phases, the dependence of identification depth on chromatographic parameters is reduced compared to earlier-generation workflows, though the extent of this effect beyond the materials tested here remains to be established (24, 25).

To test whether the observed length independence extends beyond the initial HeLa column screen, we varied matrix complexity, sample input, and gradient duration on a single stationary phase. These experiments confirmed that column length has limited impact on identification depth under most conditions; differences became apparent only at the sensitivity limits of the workflow, such as very low input amounts (<500 pg) or highly complex matrices like plasma.

Across all experimental conditions, classical chromatographic behavior was evident: shorter columns and abbreviated gradients produced broader peaks and reduced separation. However, the magnitude of these chromatographic differences did not correspond to proportional changes in identification depth or quantitative variation under the acquisition conditions used here. Taken together, this indicates that within the tested range, the downstream impact of reduced separation is smaller than in earlier LC–MS workflows, although the underlying chromatographic principles remain unchanged. The cases in which column length or gradient duration did influence performance, very low input amounts or highly complex matrices, illustrate that chromatography continues to define limits under more challenging conditions. Notably, while column length showed limited influence, stationary-phase chemistry produced distinct retention patterns despite comparable overall identification numbers.

The existence of distinct retention patterns within this convergent performance landscape highlights how column chemistry may still play a role in future analytical strategies. Our analysis of orthogonal “elution fingerprints” shows that, although bulk identification metrics have largely converged, each stationary phase still accesses complementary regions of physicochemical space. For example, EM preferentially identified early-eluting hydrophilic peptides, whereas RS favoured late-eluting hydrophobic sequences, indicating that these two chemistries preferentially sample different extremes of the retention window. PH showed characteristic aromatic interaction effects, with an enrichment of peptides containing aromatic residues. This behavior aligns with the more limited but distinct aromatic selectivity reported for phenyl phases and attributed to π–π interactions with the electron-rich phenyl ring (8, 10). These differences suggest that in modern high-throughput proteomics, column choice now serves more of a strategic role in shaping selectivity than an essential role in determining identification depth.

Mechanistically, the contrast between EM- and RS-type C18 materials, which share monomeric bonding, identical carbon load (19%), and pore size (100 Å), may reflect differences in particle size distribution rather than bonding chemistry. The EM phase exhibits a near-monosized particle population, whereas RS phases show broader size distributions (Supplemental Table S1), which can affect column bed homogeneity, local flow heterogeneity, and ultimately peak shape and separation efficiency. (7, 10). A similar shift in perspective applies to column dimensions. Classical chromatographic theory and kinetic analysis predict benefits from increased separation performance, though longer columns and smaller particles, and such strategies have indeed been shown to enhance proteome depth in conventional shotgun workflows (10, 11, 23, 26). Yet in our data, extending columns from 40 mm to 140 mm did not produce measurable improvements in peak width for most stationary phases under the steep gradient conditions used, and yielded only minimal gains in precursor and protein identifications. This observation is consistent with gradient-dominated peak width behavior, where gradient steepness rather than column plate count governs the observed peak widths. This divergence from traditional expectations suggests that, under fast DIA acquisition on modern instruments, mass spectrometric sampling capacity rather than chromatographic resolving power has become the primary determinant of identification depth, relegating column chemistry and length to tuning selectivity and peak shape within an already saturated identification regime.

In our hands, these findings establish a new framework for proteomics method development that prioritizes analytical objectives over legacy optimization assumptions. For total proteome profiling, we document a performance equivalence across different column lengths and even packing phases, providing us with unprecedented flexibility based on practical rather than analytical constraints. However, the orthogonal retention fingerprints preserve opportunities for strategic optimization when targeting specific peptide populations, aligning with emerging multiphase screening approaches (27, 28). With proteomics studies comprising many thousands of samples annually (29, 30), and the minimal performance differences between column chemistries and lengths, the priority for selection of a chromatographic column is shifted towards reproducibility and operational lifetime, thus seeking robustness over long time periods. Consistent batch-to-batch performance and extended column longevity are now as critical as selectivity for minimizing variability and costs in sustained proteomics campaigns (6), especially considering the increased throughput per day on a chromatographic system compared to only years ago. This fit-for-purpose philosophy represents a fundamental departure from the comprehensive optimization approaches that dominated separation-limited workflows (31, 32).

The scope of our findings is defined by their application to unfractionated proteome analyses acquired under standardized DIA conditions. While we systematically varied column chemistry, length, gradient duration, and sample load, other influential parameters, such as flow rate, windowing strategies, or gradient-specific optimization were kept constant to enable controlled comparisons. As a result, the relationships observed here may differ under acquisition schemes that employ tailored DIA windows, alternative MS platforms, or distinct chromatographic settings. In addition, certain specimen types may exhibit stronger dependencies on chromatographic performance than the matrices evaluated in this study. Highly dynamic-range samples such as plasma, very low-input material approaching the limits of detection, PTM-enriched peptides, or analyses performed using DDA are all contexts in which resolving power and stationary-phase properties may exert a more pronounced influence on proteome coverage (33, 34). In particular, chromatographic separation remains essential for distinguishing species that are indistinguishable by MS alone, for example positional isomers or peptides carrying posttranslational modifications at different sites, as well as for differentiating isomeric compounds with identical mass spectra or for separating diastereomeric and enantiomeric species (34, 35). Together, these considerations define the boundaries within which our conclusions apply and outline scenarios where traditional chromatographic optimization may retain greater relevance, even as its impact on modern routine high-speed DIA workflows appears increasingly constrained.

The implications of our results extend beyond column selection and may help guide the redefinition of optimization priorities across proteomics workflows. Under the fast DIA conditions used, the high MS sampling rate appears to mitigate many of the identification losses traditionally attributed to suboptimal chromatographic performance. Under these conditions, optimization priorities may shift from maximizing chromatographic performance toward MS-centric considerations such as acquisition strategy and data processing. In this setting, chromatographic optimization increasingly focuses on practical aspects such as reproducibility and column lifetime, supporting a more strategic rather than exhaustive approach to maximizing analytical success in modern high-throughput proteomics platforms.

## Data Availability

The mass spectrometry proteomics data have been deposited to the ProteomeXchange Consortium *via* the PRIDE partner repository with the dataset identifier PXD067710 and PXD072114.

reviewer_pxd067710@ebi.ac.uk

reviewer_pxd072114@ebi.ac.uk

## Supplemental data

This article contains supplemental data.

## Conflict of interest

Philipp E. Geyer is the founder and CSO of ions.bio GmbH.

## Supplemental Data

Column_screen_Supplementary_material_clean
