# Supplementary material for: Limited Impact of Column Chemistry and Length on Proteome Coverage Under High-Speed DIA
Source: Mol Cell Proteomics. 2026 Jun 23;25(7):101609. doi: 10.1016/j.mcpro.2026.101609 (PMC13400878; doi:10.1016/j.mcpro.2026.101609)
Supplement: Column_screen_Supplementary_material_clean [file mmc1.docx]

**Supplementary Material**

Supplementary table 1: Physicochemical properties of evaluated stationary phases

|  | **EM** | **RS13** | **RS15** | **C8** | **PH** |
| --- | --- | --- | --- | --- | --- |
| **Carbon load** | 19.00% | 19.00% | 19.00% | 11.00% | 17.00% |
| **Monomeric / Polymeric binding** | Monomeric | Monomeric | Monomeric | Polymeric | Polymeric |
| **Pore sizes** | 100 Å | 100 Å | 100 Å | 100 Å | 100 Å |
| **Bead size distribution (90/10)** | Close to 1 (monosized) | 1.43 | 1.47 | 1.56 | 1.6 |
| **Nominal particle size** | 1.35 μm | 1.3 μm | 1.5 μm | 1.8 μm | 1.8 μm |
| **End-capping status** | Yes | Yes | Yes | Yes | Yes |


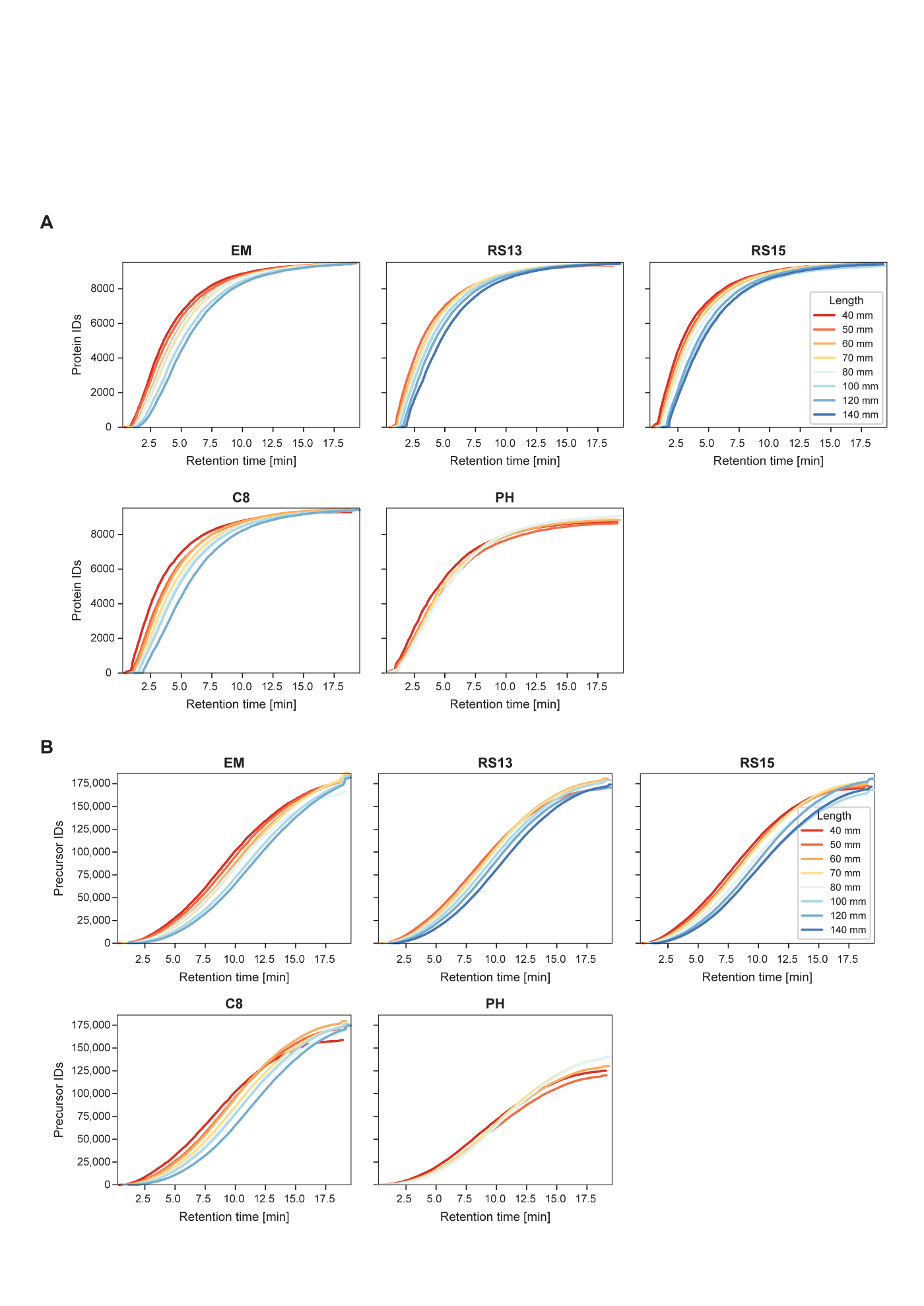


Supplementary Figure 1: **Cumulative identification profiles across column lengths.**

A: Cumulative protein identifications over retention time for each column chemistry.

B: Cumulative precursor identifications over retention time for each column chemistry.


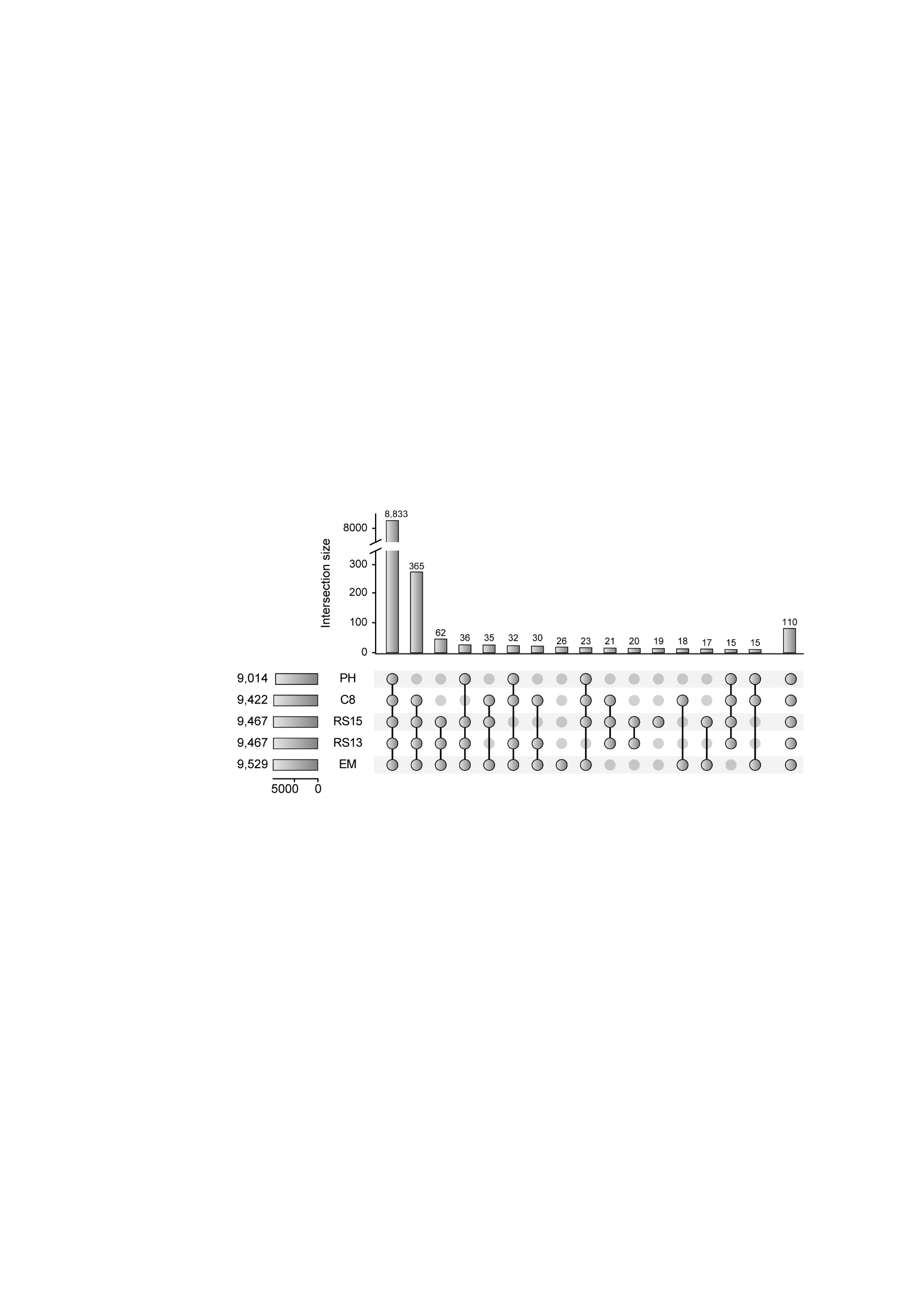


Supplementary Figure 2: **Protein group overlap across column chemistries at 70 mm.**

UpSet plot showing shared and unique protein groups between columns. Only the top intersections are shown; all remaining overlaps and unique sets are aggregated in the final bar.


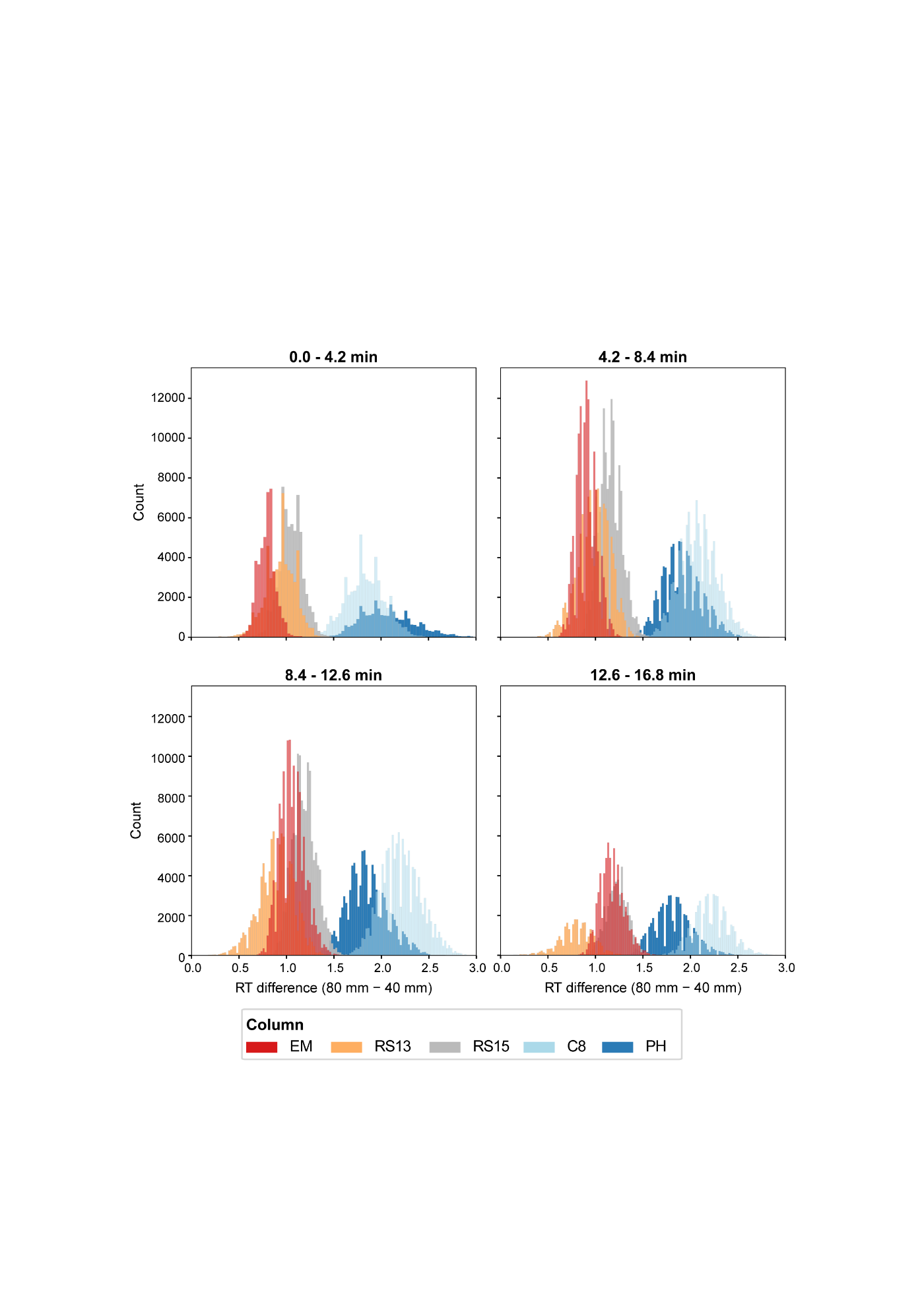


Supplementary Figure 3: Retention time difference distributions between 80 mm and 40 mm columns stratified by elution time windows.

Histograms show the distribution of retention time differences (ΔRT = RT₈₀ – RT₄₀) for precursors binned into four retention time intervals: 0.0-4.2, 4.2-8.4, 8.4-12.6, and 12.6-16.8 minutes, based on their 40 mm column retention time. Each panel represents a specific elution window, with column chemistries color-coded as indicated in the legend. Precursors eluting after 16.8 minutes are not shown.


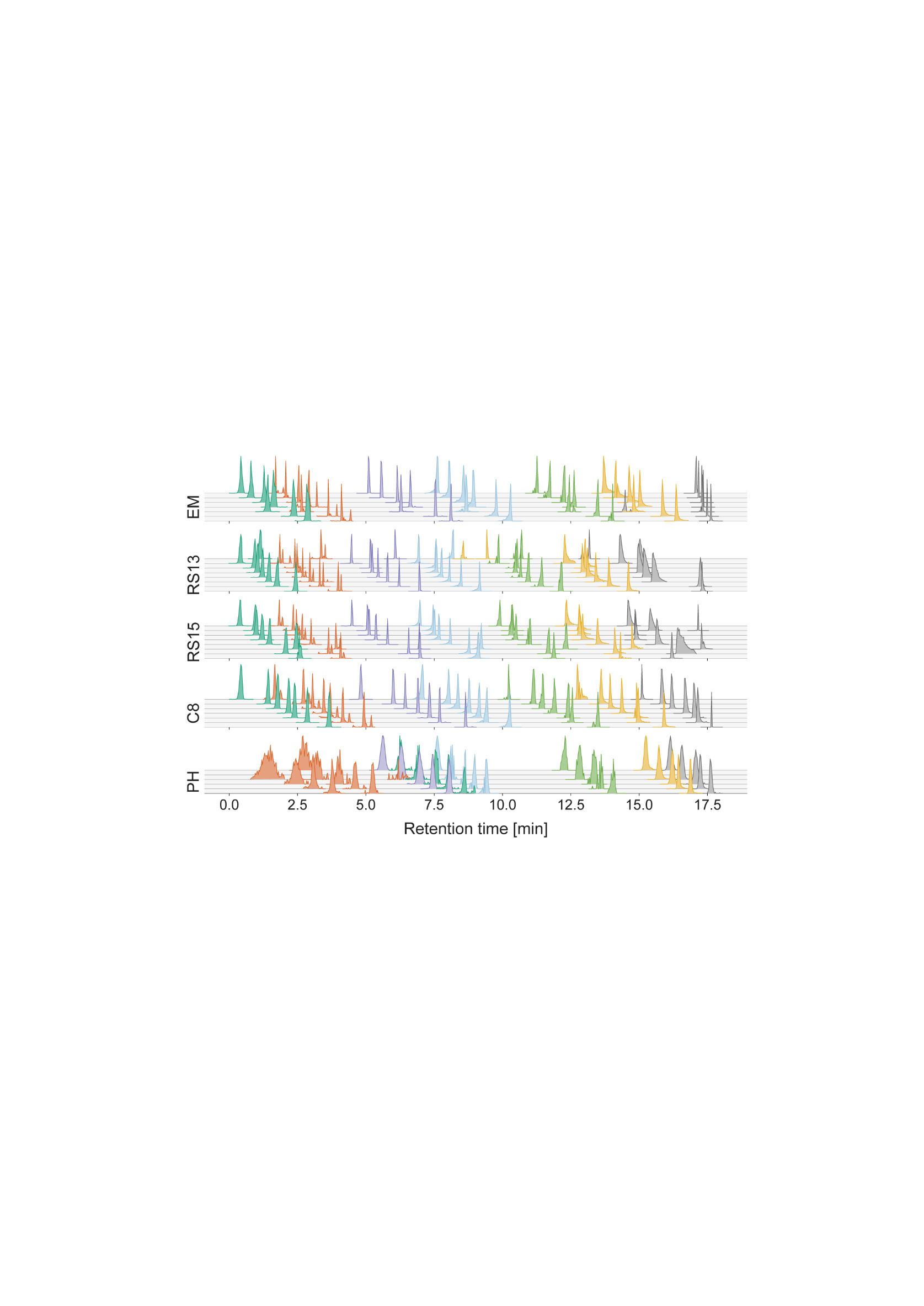


Supplementary Figure 4: Extracted ion chromatograms of GAPDH precursors across column chemistries and lengths.

Visualized replicate 3, selected precursors in retention time order: QASEGPLK2 (415.22433 m/z, dark green/turquoise), LTGMAFR2 (398.21274 m/z, orange), VGVNGFGR (403.2194 m/z, purple), GALQNIIPASTGAAK2 (706.3988 m/z, pink), LVINGNPITIFQERDPSK3 (681.0407 m/z, light green), WGDAGAEYVVESTGVFTTMEK3 (759.6842 m/z, yellow), VIHDNFGIVEGLMTTVHAITATQK4 (649.59546 m/z, grey). Precursors selected from >50 GAPDH precursors by filtering for intensity >4.0E+05 and retention time distribution. For each column material, chromatograms are arranged from top to bottom in order of increasing column length (40–140 mm, where available). All chromatograms were acquired using a 20-minute linear gradient. Note: Chromatograms represent replicate 3 for visualization. Some columns show retention time shifts (e.g., EM 80 mm with earlier elution, RS15 100 mm with later elution) that reflect column-specific variability, likely due to improper column connection by the operator or column irregularities/packing differences, as evidenced by anomalous pressure profiles.


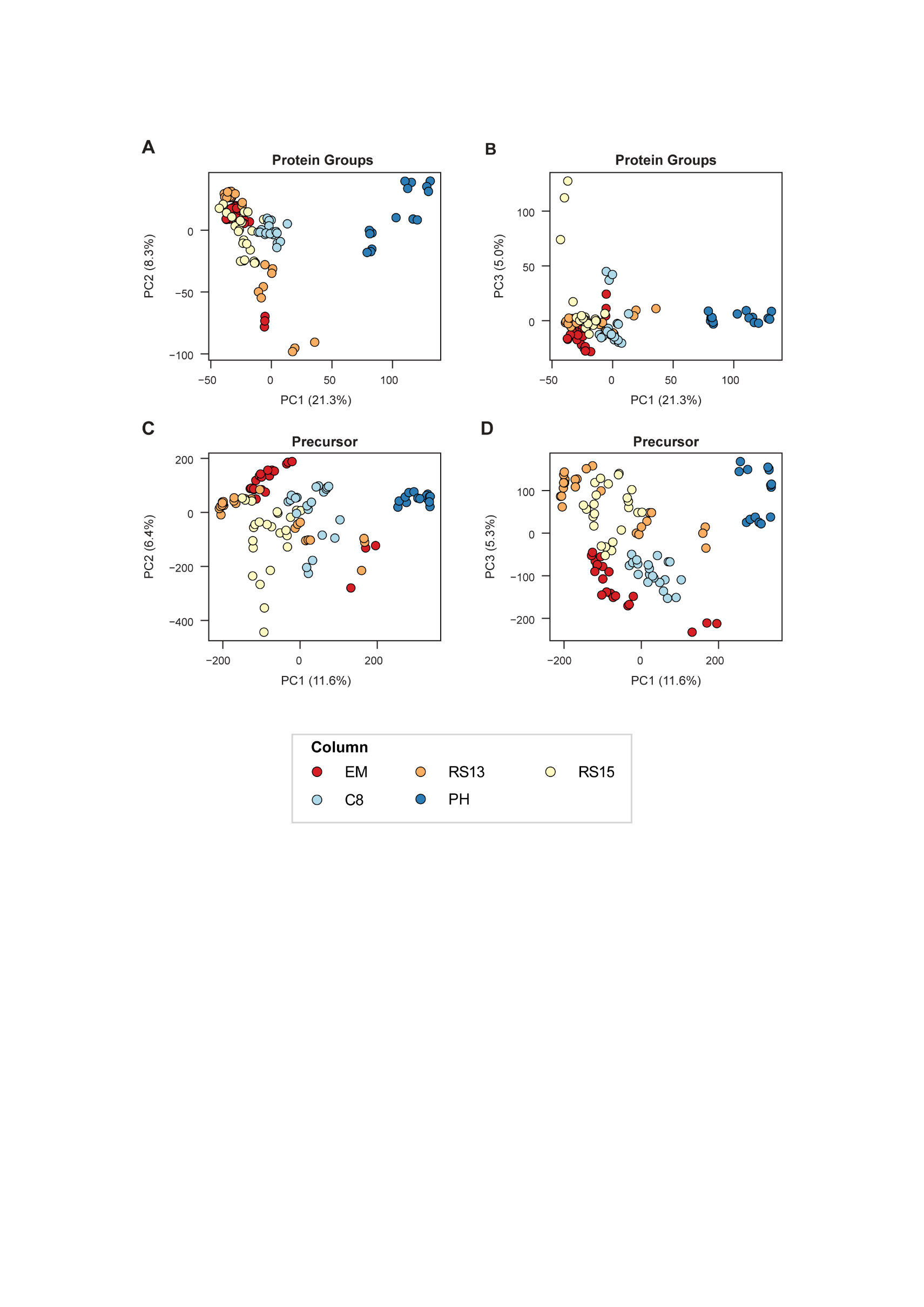


Supplementary Figure 5: Principal component analysis (PCA) of identified protein groups and precursors.

A, B: PCA of protein group intensities (PC1 vs. PC2 and PC1 vs. PC3).

C, D: PCA of precursor intensities (PC1 vs. PC2 and PC1 vs. PC3). Panel C is identical to Figure 1E and is included here for completeness.

All intensities were standardized (mean = 0, variance = 1) prior to PCA; missing values were imputed using k-nearest neighbor (k = 5).


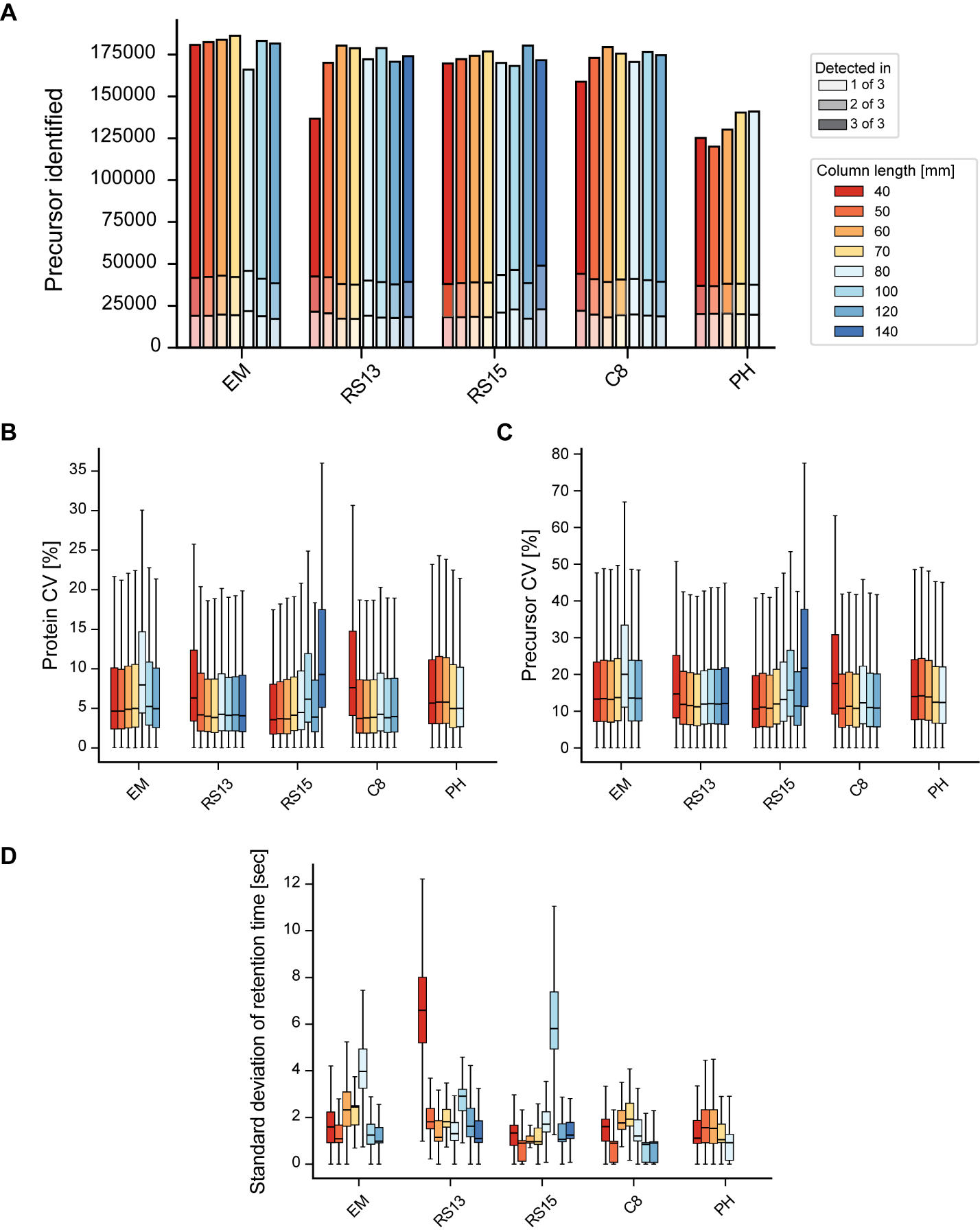


Supplementary Figure 6: Quantitative reproducibility across column chemistries and lengths.

(A) Number of precursors detected in one, two, or all three replicates (stacked bars) for each column chemistry and length. (B) Protein-level coefficient of variation (CV) distributions across replicates. (C) Precursor-level CV distributions across replicates. (D) Standard deviation of retention times across replicates. All panels show data grouped by column chemistry with individual lengths color-coded (40-140 mm).


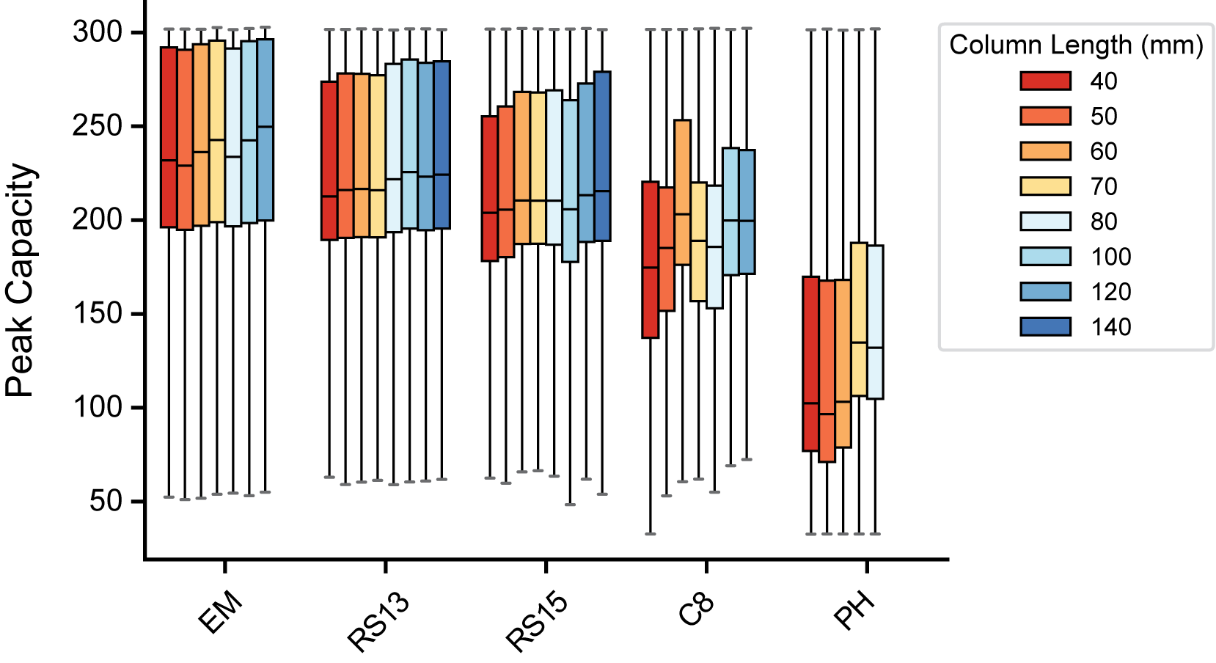


Supplementary Figure 7: **Estimation of chromatographic peak capacity across column chemistries and lengths.**

Peak capacity was calculated as n_c_ = 1+(t_g_ / 1.7 * w_h_), where t_g_ is the gradient time of 20 minutes and w_b_ the peak width at baseline. Boxes show the distribution of estimated peak capacities per column chemistry and length. Peak capacity was calculated individually for each identified precursor using the FWHM reported by DIA-NN.


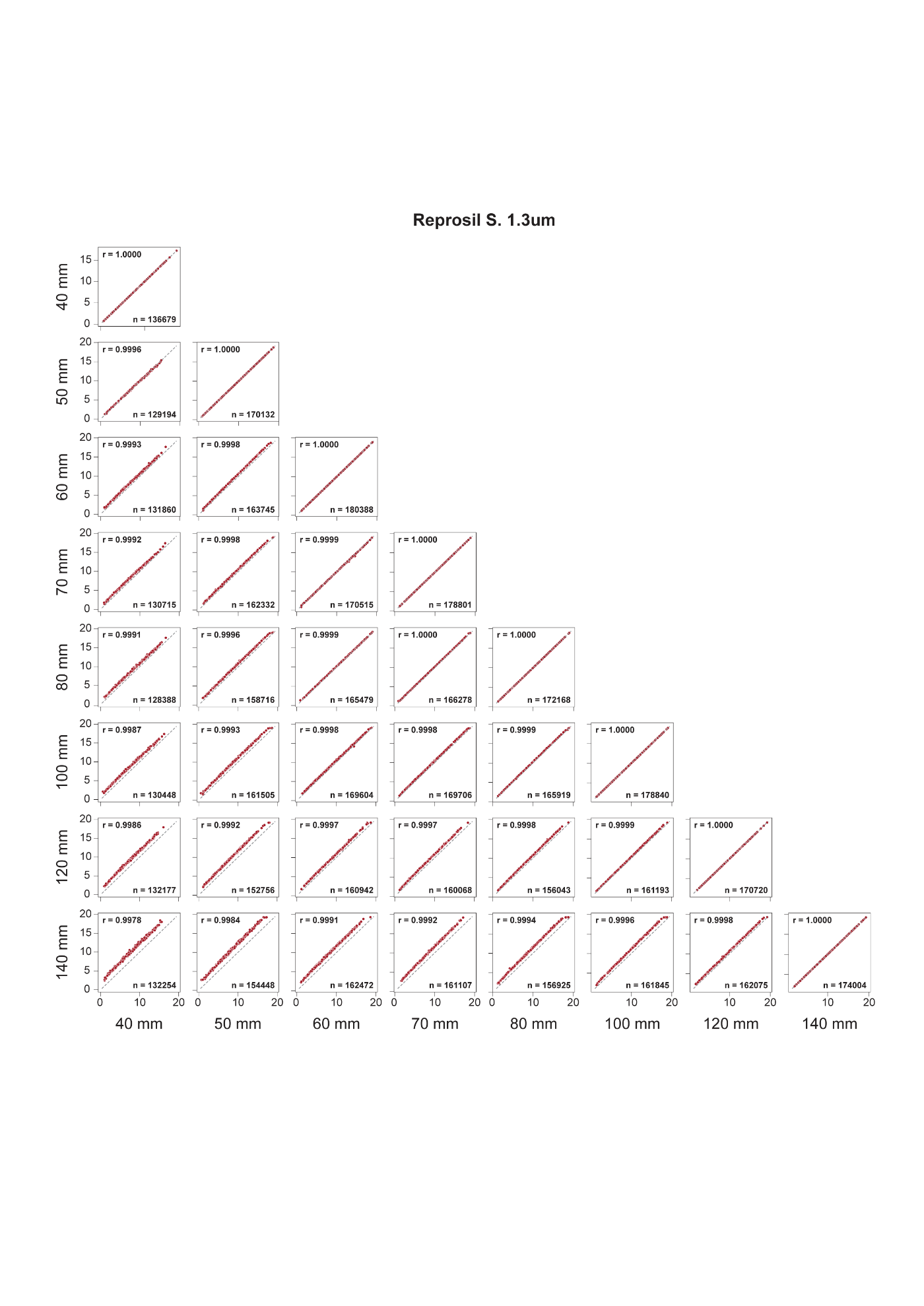


Supplementary Figure 8: **Retention time correlations across column lengths for RS13.**

Pairwise retention time correlations between all column lengths (40–140 mm) for RS13; only 0.2% of all data points are shown.


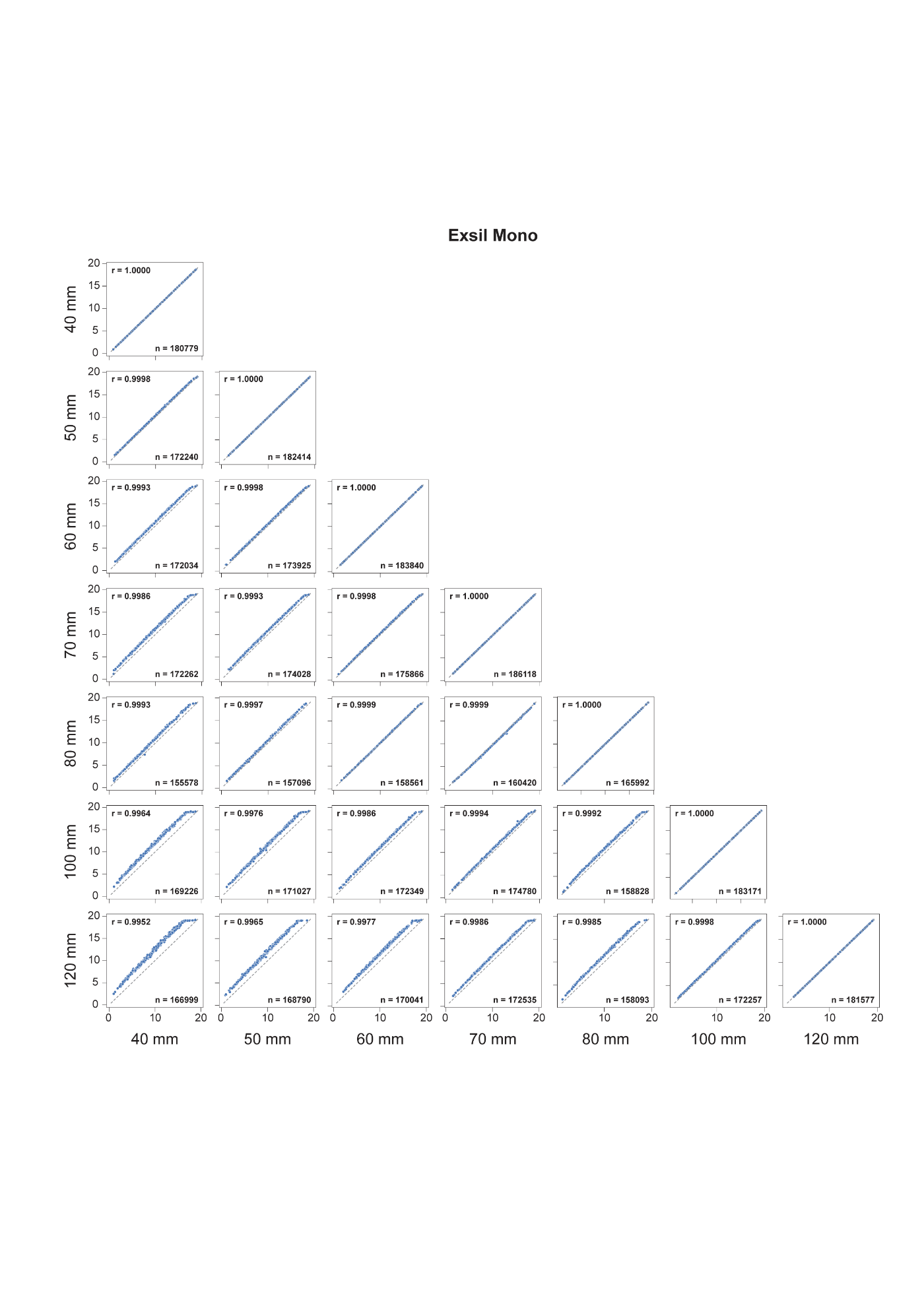


Supplementary Figure 9: **Retention time correlations across column lengths for EM.**

Pairwise retention time correlations between all column lengths (40–120 mm) for EM; only 0.2% of all data points are shown.


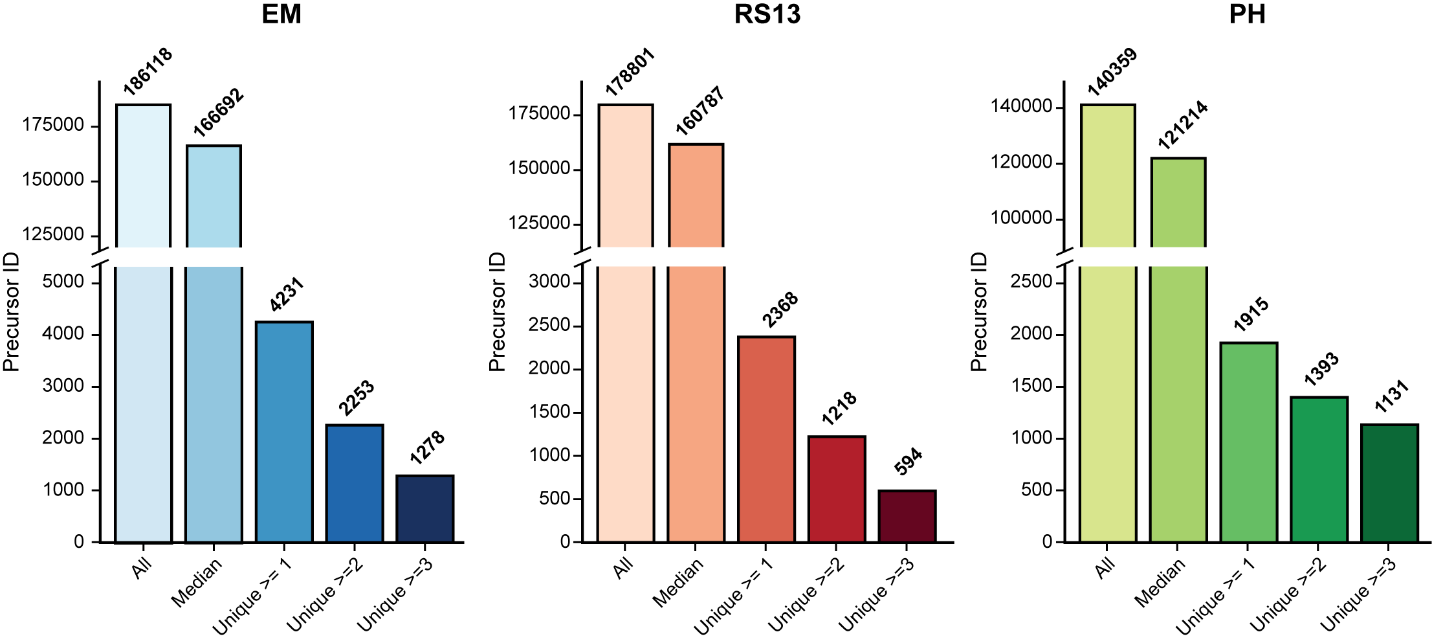


Supplementary Figure 10: **Filtering cascade for column-unique precursors (70 mm columns)**

Bars illustrate the sequential filtering of precursor identifications for the EM, RS13, and PH columns. The x-axis shows the progression from all precursors identified in any replicate (All) to the median number per replicate (Median), and to precursors detected exclusively with a given stationary phase in at least one, two, or all three replicates (Unique ≥1, Unique ≥2, Unique ≥3). Numbers above bars indicate the remaining precursors after each step. The figure demonstrates the increasing stringency of the reproducibility filter.


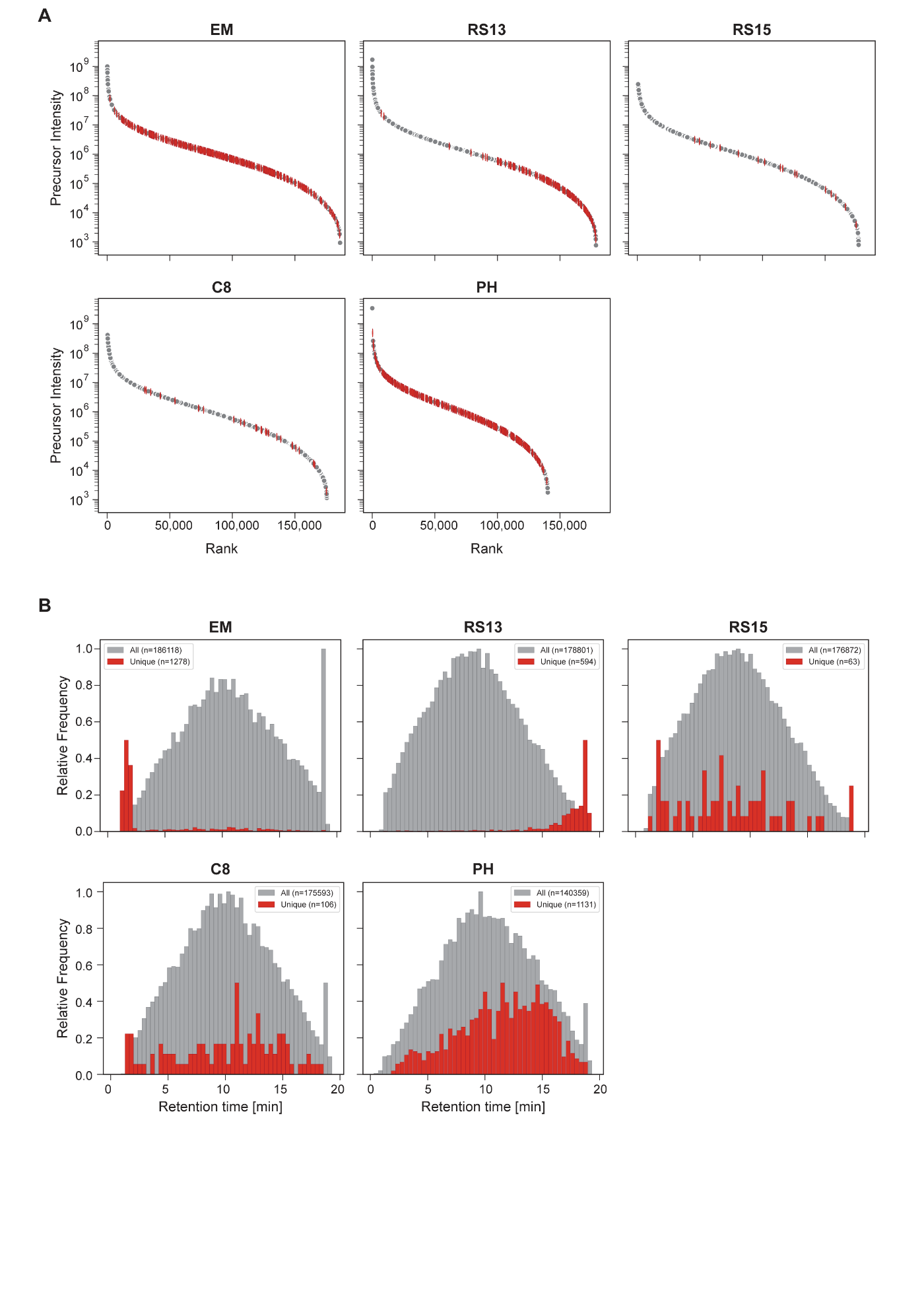


Supplementary Figure 11: **Unique precursor analysis for all column types.**

A: Rank abundance plots for each column showing all precursors (grey) and unique precursors (red). Only 1% of grey dots is shown and 25% of the red dots.

B: Retention time distributions for each column. Distribution of all precursors is normalized to 1 and distributions of the unique precursors are normalized to 0.5.


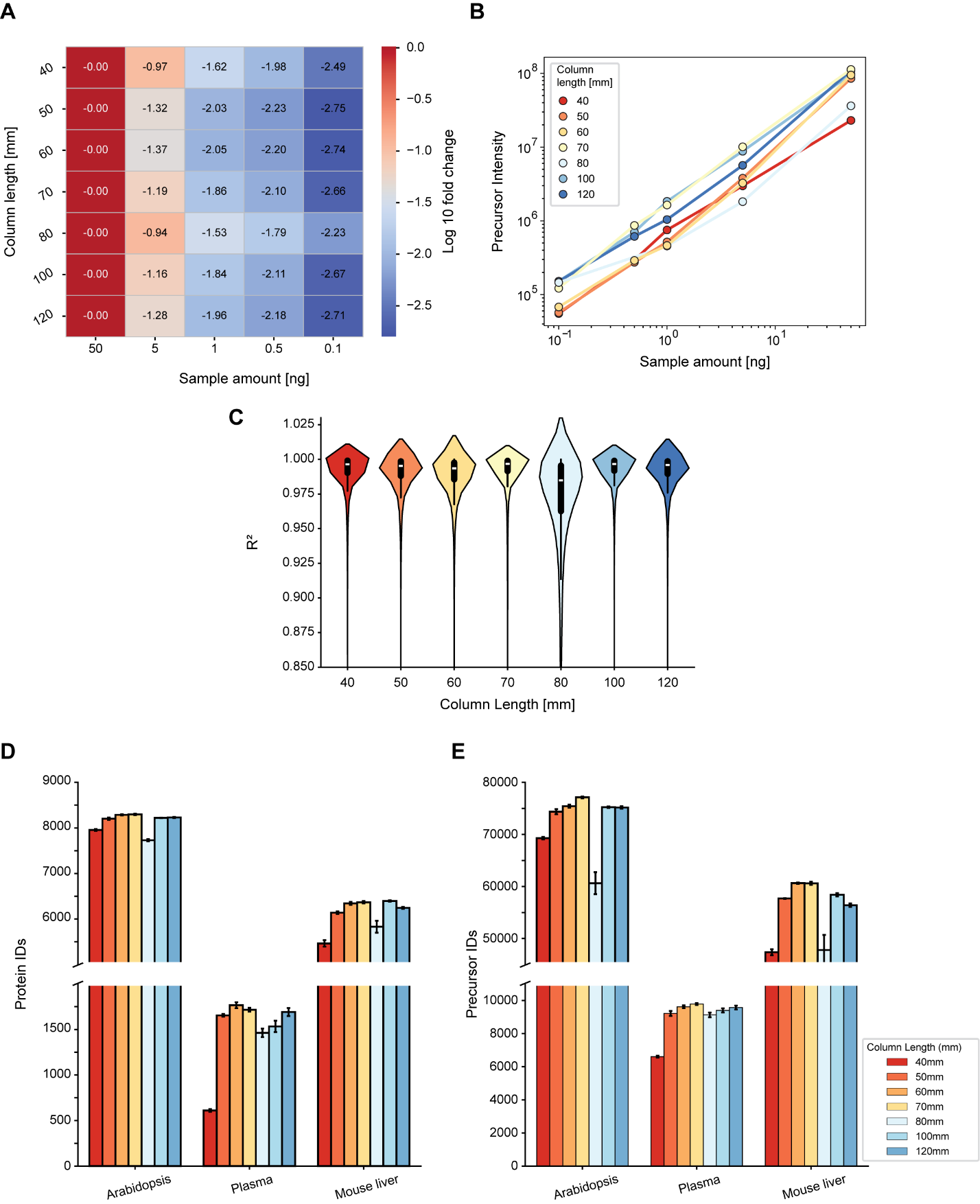


Supplementary Figure 12: Additional evaluation of column performance across lengths and sample types. (A) Heatmap of median log₁₀ fold-changes in precursor intensities across sample input amounts (0.1–50 ng) for all tested column lengths (40–140 mm). Values represent the median deviation from the expected fold-change relative to 50 ng. (B) Log–log plot of precursor intensities for the GAPDH VGVNGFGR2 precursor across sample input amounts, shown for all column lengths. Lines illustrate the linear dynamic response of each column. (C) Distribution of R² values from linear regression of precursor intensities across the dilution series for each column length, illustrating quantitative linearity across all detected precursors. (D) Protein group identifications obtained from three different sample types (Arabidopsis, human plasma, and mouse liver) across all column lengths. Bars represent mean ± standard deviation (n=3). (E) Precursor identifications for the same sample types and column formats as in panel D. Bars represent mean ± standard deviation (n=3).


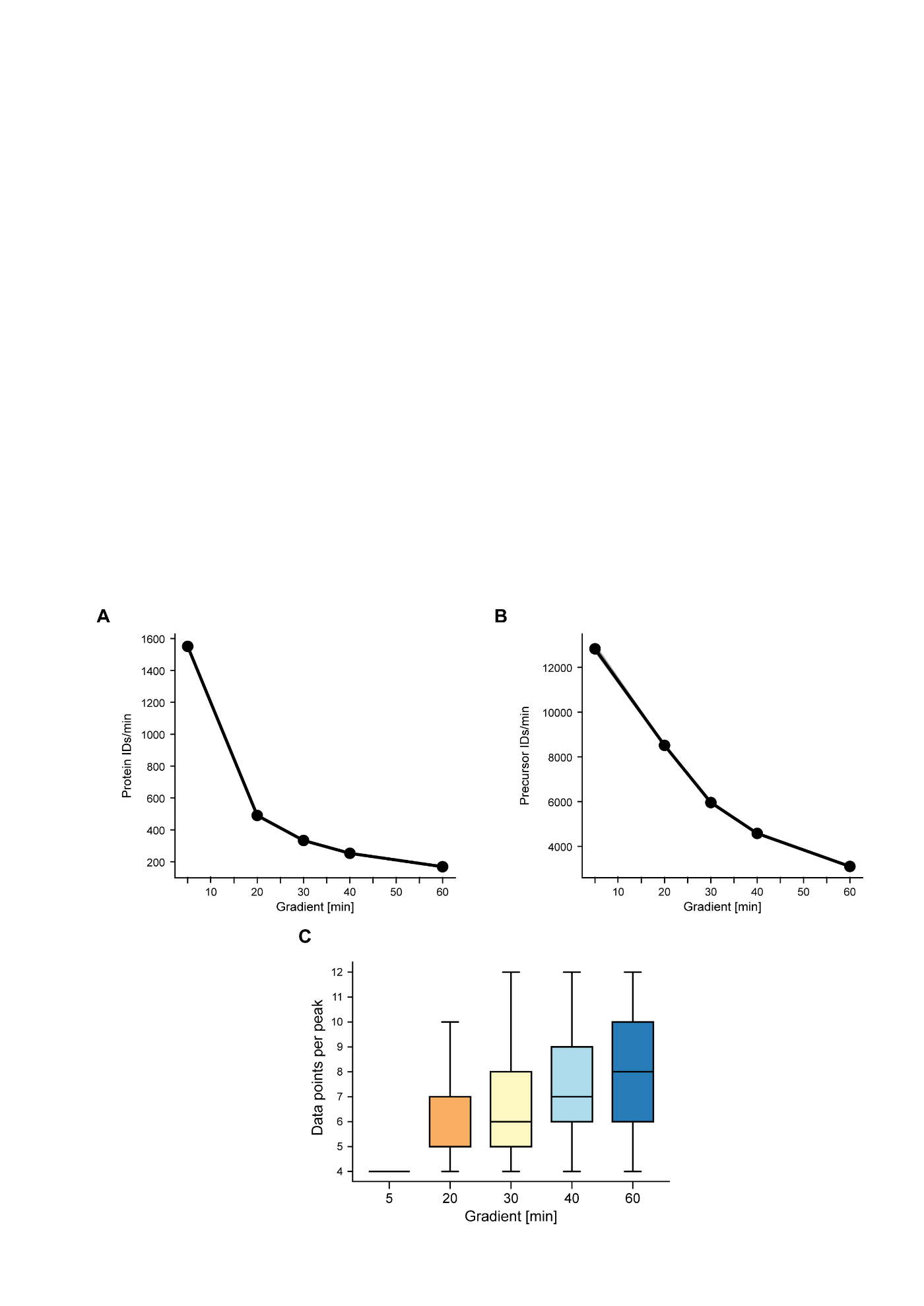


Supplementary Figure 13: Impact of gradient length on identification rate and chromatographic sampling. (A) Protein group identifications normalized to gradient duration (protein IDs per minute) across gradient lengths from 5 to 60 minutes. (B) Precursor identifications normalized to gradient duration (precursor IDs per minute) for the same gradients. Both panels illustrate the decline in identification rate per unit time as gradients are extended. (C) Distribution of data points per chromatographic peak across gradient lengths, shown as boxplots. Longer gradients yield increased sampling density per peak.
